# Influence of Furfuryl Alcohol on the Cross-Linking Process of Selected Industrial Phenol-Formaldehyde Resins

**DOI:** 10.3390/polym18202513

**Published:** 2026-10-14

**Authors:** Maciej Gubernat, Piotr Jelen, Jakub Marchewka, Aleksandra Benko, Ryszard Wielowski, Marcel Zambrzycki, Krystian Sokolowski, Aneta Fraczek-Szczypta

**Affiliations:** 1 Faculty of Materials Science and Ceramics, AGH University of Krakow, Av. Mickiewicza 30, 30-059 Krakow, Poland; 2 Academic Centre for Materials and Nanotechnology, AGH University of Krakow, Av. Mickiewicza 30, 30-059 Krakow, Poland

**Keywords:** phenol-formaldehyde resin, furfuryl alcohol, methylene bridges, curing behavior, carbon-carbon composite precursor

## Abstract

Furfuryl alcohol (C_5_H_6_O_2_, FA) is commonly used as a modifier for phenol-formaldehyde (PF) resins and has been reported to influence their curing behavior and processing performance. Comparative analysis of commercial resin systems with and without FA suggested that the presence of FA influences the distribution of methylene bridge linkages formed during curing. FT-IR spectra indicated an increased contribution of para-para linkages in FA-containing resins, with this tendency becoming more pronounced at elevated temperatures. MAS-NMR spectra revealed differences in local methylene bridge environments, supporting the conclusion that FA influences the organization of the cross-linked network. XPS analysis revealed differences in the chemical structure of the cured resins, including a higher contribution of oxygen-containing functional groups in the FA-modified systems. STA-FT-IR results showed that FA-containing resins exhibited a more gradual curing process accompanied by a more uniform release of volatile products. Dynamic viscosity measurements demonstrated a substantial reduction in viscosity after FA addition, from 1051 to 228 mPa s for the F110-based resin system and from 1104 to 144 mPa s for the MC-05-based system. The results suggest that FA influences the cross-linking pathway, curing behavior and processing properties of PF resins. Improved understanding of these effects may support advanced PF-based composites manufacture.

## 1. Introduction

Phenol-formaldehyde (PF) resins are widely employed on an industrial scale in applications ranging from foundry and polymer composites [1] to the production of hard carbons for batteries, friction materials, abrasive materials, ablative materials, rubbers and adhesives [2,3,4,5,6]. Among the products containing PF resins, construction insulation materials, cutting and friction tools, brake pads, special products such as composite elements of rocket engines, heat shields and many others [7,8,9,10,11] can be listed. The diversity of applications contributes to the use of PF resins in various manufacturing technologies, including composite material production by pultrusion, additive manufacturing, filament winding, resin transfer molding and hand lay-up processes. Because of the diverse applications of PF resins, developing core knowledge regarding their processing and specialized use is of global significance [12,13,14,15,16].

There are two principal types of phenol-formaldehyde resins: resols and novolacs. Their structures and curing behavior are strongly influenced by the conditions used during synthesis, particularly the pH and the formaldehyde-to-phenol molar ratio. Resols are generally synthesized under basic conditions using alkaline catalysts, such as NaOH or Ba(OH)_2_, with formaldehyde present in molar excess relative to phenol. These conditions promote the formation of hydroxymethyl groups, which provide reactive sites for subsequent condensation and development of a three-dimensional cross-linked network. Novolacs, in contrast, are typically synthesized under acidic conditions with a lower formaldehyde-to-phenol ratio. They predominantly consist of phenolic oligomers with limited hydroxymethyl functionality and generally require an additional curing agent, such as hexamethylenetetramine, to form a cross-linked thermoset network. Thus, both resols and novolacs can ultimately form thermoset materials, but they differ in the mechanism and conditions required to generate the cross-linked structure. The resol resins investigated in the present study were synthesized under basic conditions, using NaOH or Ba(OH)_2_ as alkaline catalysts [17,18]. Resol resin cross-linking occurs through polycondensation reactions accompanied by water release. The literature describes three main pathways: formation of para-para (p-p) methylene bridges through reactions involving para-hydroxymethyl groups, formation of ortho-ortho (o-o) methylene bridges through reactions involving ortho-hydroxymethyl groups and condensation between hydroxymethyl groups leading to the formation of ether linkages [19]. Resol resins exhibit gradual cross-linking even at ambient temperatures, resulting in a finite shelf life [20].

**Figure 1:**
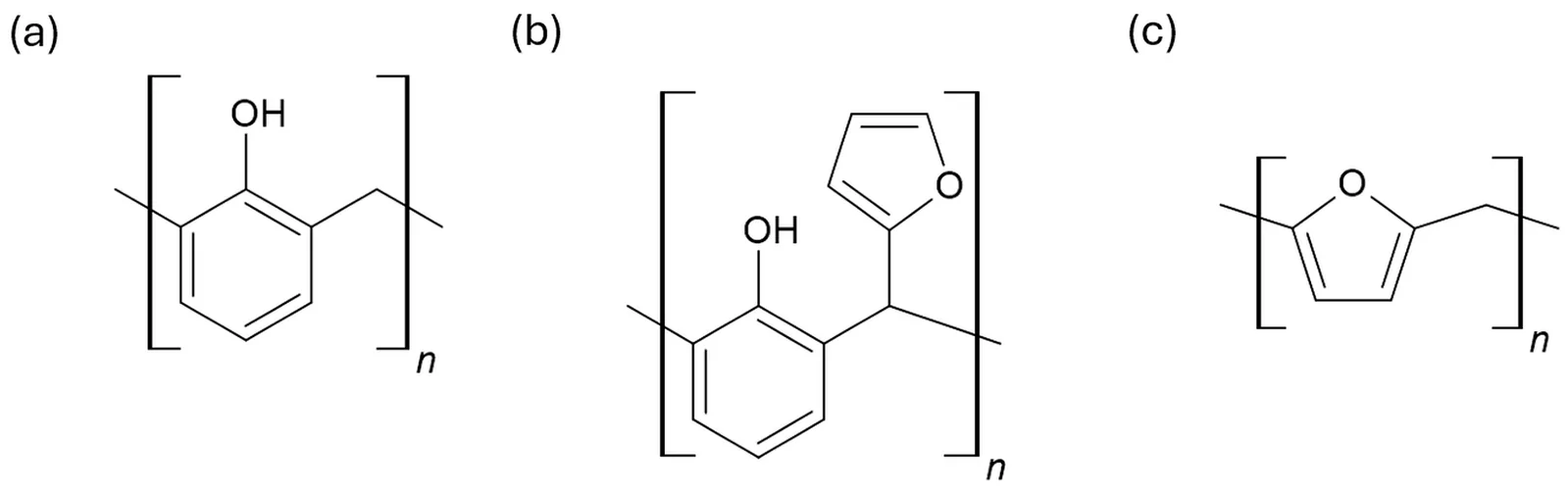
Reaction products of (**a**) phenol with formaldehyde, (**b**) phenol with furfural, (**c**) furfuryl alcohol with formaldehyde (only an exemplary linkage route between the specific moieties is illustrated).

Furfuryl alcohol (C_5_H_6_O_2_, FA) is obtained by the hydrogenation of furfural (aldehyde) [21]. Acid-catalyzed homopolymerization of FA yields oligomers, primarily dimers and trimers, linked by methylene bridges between furan rings. The resinification process of FA is highly exothermic, requires temperature control and is carried out to obtain the desired viscosity of the obtained resin. Under appropriate conditions, FA forms copolymers with formaldehyde, furfural and phenol (Figure 1). Hence, the addition of FA should affect the cross-linking process of the resol resin [6,21].

The incorporation of FA into PF resin systems is of considerable technological interest. In industrial processing of PF-based composites, the curing behavior of the resin strongly influences the manufacturing efficiency and the quality of the final product. Excessively rapid cross-linking and uncontrolled evolution of volatile by-products may result in defects such as porosity, void formation or fiber misalignment. FA has been reported to modify the curing behavior of thermosetting systems and may contribute to improved process stability and defect reduction [22]. However, despite its practical importance, the mechanisms by which FA influences the cross-linking reactions of PF resol resins remain insufficiently understood. In particular, the relationship between FA incorporation, methylene bridge formation and curing behavior has not yet been fully explained.

Our previous research [22] demonstrated that incorporating FA into resol matrices for pultruded carbon fiber composites significantly enhances the cross-linking process. By slowing cross-linking kinetics and broadening the reaction temperature range, FA facilitated the release of a substantial quantity of gaseous by-products at lower temperatures during gelation, before the resin solidified. These changes resulted in a reduced risk of defects in the composite, such as pores in the matrix and fiber deviation from the proper axis in the product. FA improved the repeatability of the mechanical properties of carbon-resol composites. The specialized use of resol resins in fibrous ablative and carbon-carbon composites, vital for the burgeoning space and defense industries worldwide, underscores the crucial nature of this research.

The aim of this study is to provide a more comprehensive understanding of the influence of FA on the cross-linking behavior of industrial PF resol resins. In contrast to studies focused primarily on the general curing behavior of model PF systems, the present work compares industrial resin formulations with and without FA and examines their structural and curing evolution using complementary FT-IR spectroscopy, MAS-NMR spectroscopy, XPS, simultaneous thermal analysis (STA) coupled with FT-IR and rheological measurements. Particular attention is given to changes in the distribution of methylene bridge structures, the evolution of oxygen-containing functional groups, the temperature-dependent development of the curing process and viscosity. This integrated approach allows the effects of FA to be considered in relation to both the chemical structure and the processing behavior of the resin. The results provide new insight into the role of FA in modifying the cross-linking pathway of industrial PF resols and may contribute to more controlled processing and curing of FA-containing resin systems, particularly where reproducibility and control of resin flow and cross-linking are important.

## 2. Materials and Methods

### 2.1. Materials

Two commercial resol resins were selected for testing: old (Resin 1) and new generation (Resin 2), of which Resin 2 is more environmentally friendly due to its lower free formaldehyde content. Resin 2 in its commercial variant contains 30 percent of furfuryl alcohol. The compositions and selected physicochemical properties of the two commercially available resins, based on the manufacturer’s data, are given in Table 1 and Table 2. Resin 1 (F-110) has a phenol-to-formaldehyde molar ratio of 1.00:1.29 and is manufactured through the following stages: raw material charging (phenol, catalyst and formaldehyde solution), condensation to the specified water content tolerance (0.3–1.5 mL of water per gram of the resin), distillation to remove water from the resin, and subsequent dissolution of the resulting soluble, partially condensed resin in ethanol for partial adjustment of the final resin viscosity. Resin 2 (MC-05) has a phenol-to-formaldehyde molar ratio of 1.00:1.40 and is manufactured through the following stages: raw material charging (phenol, catalyst and formaldehyde solution), time-controlled condensation, neutralization to stabilize the resin, distillation to remove water from the resin, and subsequent dissolution of the resulting soluble, partially condensed resin in furfuryl alcohol for partial adjustment of the final resin viscosity. The condensation step was performed under the manufacturer’s specified processing conditions.

To evaluate the impact of FA on resin cross-linking, the manufacturer provided resin samples made exclusively for these tests: Resin 1 modified with FA designated as Resin 3 and Resin 2 without FA designated as Resin 4 (Table 2). To investigate the effect of FA on the cross-linking reaction mechanism of selected PF resins, Resins 1–4 were analyzed using the following techniques: FT-IR at room temperature (Diffuse Reflectance Spectroscopy—DRS spectra), FT-IR during heating (Diffuse Reflectance Infrared Fourier Transform Spectroscopy—DRIFTS spectra), Magic-Angle Spinning Nuclear Magnetic Resonance (MAS- NMR) and X-ray photoelectron spectroscopy (XPS). Furthermore, to demonstrate the significance of FA on practical processing aspects of the investigated commercial resins, such as technological parameters and the emission of by-products generated during the cross-linking reaction, the results of dynamic viscosity measurements and Simultaneous Thermal Analysis with FT-IR gas analysis (STA-FT-IR) for Resins 1 and 2 are presented.

### 2.2. Methods

#### 2.2.1. FT-IR

FT-IR at room temperature (DRS) and in situ FT-IR studies (DRIFT) were performed using a Bruker Vertex 70v vacuum spectrometer (Bruker Optik GmbH, Ettlingen, Germany) equipped with a Harrick Scientific Praying Mantis high-temperature chamber.

In the DRS measurements, the investigated resins were analyzed directly in the viscous state and the samples were prepared similarly to the samples for the DRIFTS measurements. A small amount of each resin was placed on an aluminum foil substrate to form a very thin film (an automatic pipette was used each time). This approach enabled the resins to be examined without dilution or incorporation into a solid matrix, which would be impractical for these viscous liquid samples.

DRIFTS measurements were carried out for each resin during continuous heating from room temperature to 250 °C at a heating rate of 10 °C min^−1^. Each recorded spectrum was obtained by co-adding 64 scans collected over approximately 1 min. Because of the applied heating rate, each spectrum represents an averaged measurement across approx. 10 °C interval, rather than the sample’s instantaneous state at a single temperature. The temperature assigned to each spectrum corresponds to the average temperature of the acquisition window. Spectra were collected in the range of 4000–400 cm^−1^ with a spectral resolution of 4 cm^−1^. Consequently, the DRIFTS configuration was used to follow the temperature-dependent spectral evolution and general structural trends during curing, whereas rapid kinetic events occurring within narrower temperature intervals were not interpreted as independently resolved processes.

#### 2.2.2. MAS-NMR

The solid-state ^13^C CP MAS experiments were performed on the BRUKER Avance III 400 WB spectrometer (Bruker BioSpin GmbH, Rheinstetten, Germany), operating at 400.19 MHz for ^1^H and 100.63 MHz for ^13^C, respectively. Spectra were acquired with an MAS probe head using 4-mm ZrO_2_ rotors. A sample of native glycine was used for setting the Hartmann–Hahn condition and the sample of adamantane was utilized as a chemical shift external reference (38.48 ppm).

Spectra were recorded with a MAS frequency of 8000 Hz and a proton 90° pulse of 3.0 μs in length and a contact time set to 2 ms with a ramped CP pulse. The repetition delay was 4 s and the spectral width was 50 kHz. 1024 or 2048 scans per FID were accumulated with a time domain size of 4 K data points, with SWf-TPPM decoupling sequence applied during acquisition time. All spectra were acquired and processed using the TopSpin 2.1 program.

#### 2.2.3. XPS

XPS measurements were performed using a PHI VersaProbe X-ray photoelectron spectroscope working with a monochromatic Al Kα X-ray source (1486.6 eV). The dual-beam compensation with Ar^+^ and low-energy electrons was used to ensure charge neutrality on the surface of the samples. The photoelectron take-off angle was set to 45°. All spectra were referenced to the C1s peak at a binding energy of 285.0 eV. During measurements, the pressure in the chamber was maintained below 3 × 10^−7^ Pa. The resins subjected to XPS analysis were prepared via a heat-curing process in an oven with a heating rate of 10 °C min^−1^ to 250 °C and curing for 5 min at 250 °C. After the treatment, the samples were transferred to the spectrometer chamber. Fitting of spectra was performed using PHI MultiPeak software (v.9.9.3), with a background subtraction applied via the Shirley method.

#### 2.2.4. STA-FT-IR

STA measurements (STA 449 F3 Jupiter, NETZSCH, Selb, Germany), which combine thermogravimetry (TGA) with differential scanning calorimetry (DSC), were combined with a real-time FT-IR (Tensor 27, Bruker with a liquid nitrogen-cooled mercury cadmium telluride detector) analysis of volatiles released from the samples. The samples were placed inside alumina crucibles and heated to 250 °C, under a protective flow of nitrogen (50 mL min^−1^) at a rate of 10 °C min^−1^. A measurement performed on an empty crucible was used for baseline correction, while FT-IR background was measured by collecting the signal from the chamber before the heating was initiated. At least 3 separate measurements were recorded for each sample type and the presented data are representative curves for each material.

The results obtained were analyzed using Opus ver. 7.2 (FT-IR) and Proteus ver. 6.1 (STA) software. Identification of gaseous products was conducted via EPA/NIST standard reference database series 35 from Bruker. Upon identification of a certain product, characteristic spectral bands that did not overlap with the rest of the products were selected and their intensity was plotted against the temperature. These were: 1014 cm^−1^ for furfuryl, 1178 cm^−1^ for methane, 1602 cm^−1^ for phenol, 1650 cm^−1^ for water, 2168 cm^−1^ for CO and 2300 cm^−1^ for CO_2_. To allow direct comparison between the two sample types, the obtained curves were divided by the sample masses, so that the presented data show the release per 1 mg of raw sample. Thus, temperature-resolved release curves of certain compounds were obtained. All the graphs presented based on the obtained data were prepared using OriginPro 2026b software.

#### 2.2.5. Rheological Behavior Analysis

Rheological characterization was carried out on a modular compact rheometer (MCR 302, Anton Paar GmbH, Graz, Austria), equipped with a parallel-plate geometry (PP50; 50 mm diameter), at a standard measuring gap of 1 mm. Prior to each measurement, samples were equilibrated at the starting temperature for 5 min (Peltier-based temperature control). The shear-rate dependence of viscosity was assessed at 30 °C, over a shear-rate range of 1–500 s^−1^. All measurements were performed in triplicate and data acquisition and evaluation were carried out using RheoCompass software version 1.32.258 (Anton Paar).

## 3. Results

### 3.1. FT-IR Room Temperature Analysis

It should be noted that the studied materials were commercially available and the manufacturer provided experimental resin compositions rather than model systems synthesized under laboratory-controlled conditions. Therefore, the comparison between Resin 1 and Resin 2 cannot be treated as an isolated effect of furfuryl alcohol, as these commercial resins differ not only in FA content but also in composition details, including the type of alkaline catalyst. To reduce this limitation, the discussion of the FA effect is primarily based on paired comparisons within the same resin family: Resin 1 vs. Resin 3 and Resin 4 vs. Resin 2. In these pairs, the presence or absence of FA is treated as the main difference in the composition of the resins provided by the manufacturer. The comparison between Resin 1 and Resin 2 is used only to discuss differences between commercial compositions.

**Figure 2:**
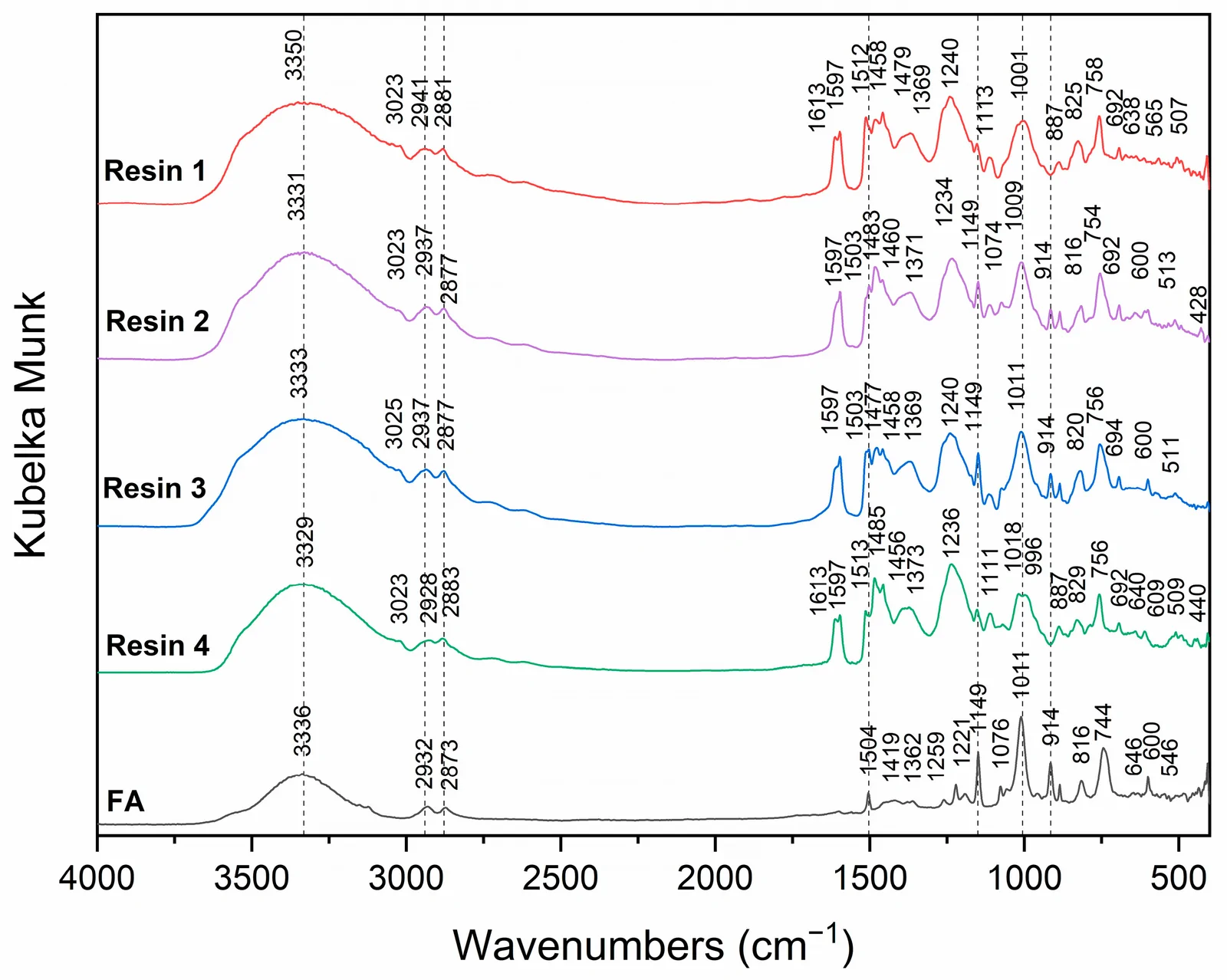
Room temperature DRS spectra of studied Resins 1–4 and FA.

Spectroscopic analysis begins by examining at room temperature, in order to identify the main spectral differences between the studied resin systems and to distinguish the bands related to FA. FA has several typical bands by which it can be recognized, as indicated by the dashed lines in Figure 2. The band at approximately 3340 cm^−1^ is responsible for O-H stretching vibrations in the C-OH system. The bands at 2932 and 2873 cm^−1^ are responsible for the stretching vibrations of the C-H groups. At 1504 cm^−1^, there is a band that can be attributed to C=C stretching vibrations in the furan ring, while C-O (C-O-C) vibrations in the same ring are visible at 1149 cm^−1^. At 1419 and 1362 cm^−1^, there are bands from the wagging vibrations of the CH_2_ groups. The most intense band in the spectrum, at 1011 cm^−1^, comes from the C-O stretching vibrations in the hydroxymethyl moiety. At 914 cm^−1^, there is a band responsible for the presence of O-H bending vibrations in the CH_2_OH moiety connected to the heterocyclic ring.

All resin samples exhibit bands at 1504, 1149, 1011 and 914 cm^−1^, characteristic of PF resins, albeit with different intensities, due to differences in their composition [19]. The only distinctive differences, resulting from the reaction of alcohol with the resin, are visible in the form of increased intensity of the band at approximately 1612 cm^−1^, which will be explained later in the text.

**Figure 3:**
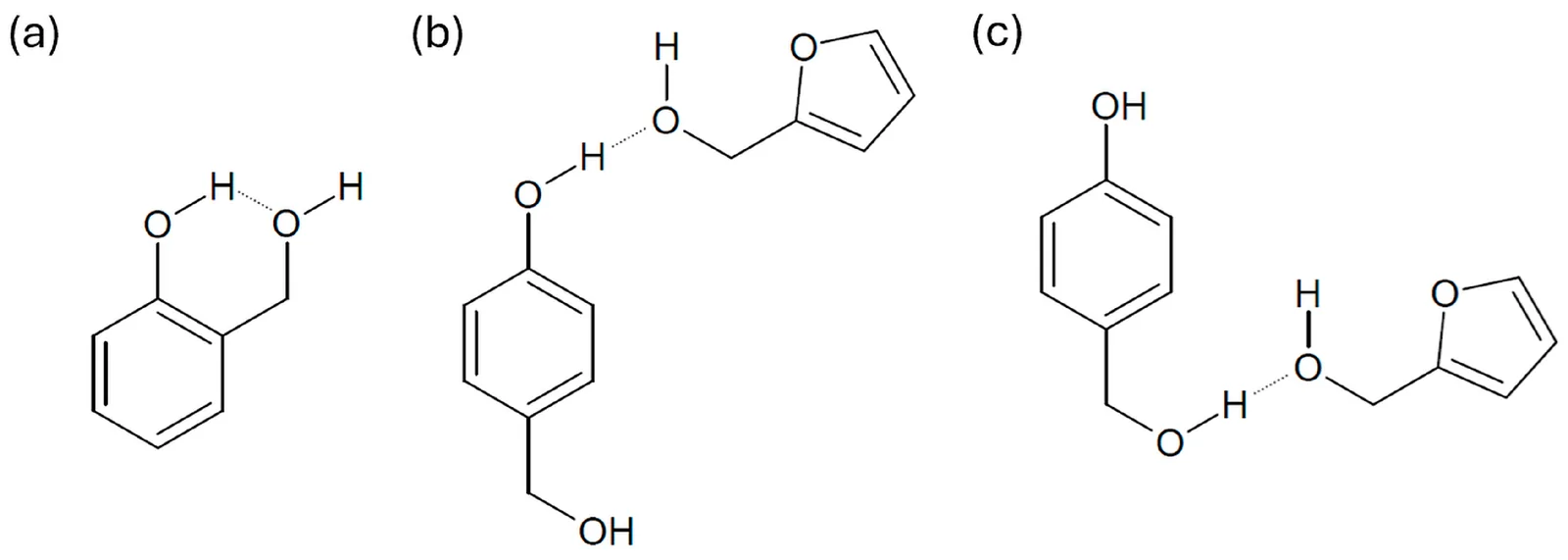
Possible different hydrogen bonds: (**a**) intramolecular, (**b**,**c**) intermolecular.

The analyzed samples of Resin 1 and Resin 4, without FA, have very similar spectral features. Both are characterized by an intense band at 3340 cm^−1^, which originates mainly from the stretching vibrations of the O-H group in phenols and/or hydrogen-bonded hydroxyl species. For resins containing FA (Resin 2 and Resin 3), there is a clear increase in the band intensity at approximately 3620 cm^−1^, as well as a slight increase at 3540 cm^−1^. These changes originate from the inter- and intramolecular hydrogen bonds, respectively. These features suggest changes in the hydrogen bonding environment after FA addition. Such changes may result from interactions between FA hydroxyl groups and phenolic or hydroxymethyl groups in the resin, as schematically presented in Figure 3. However, due to the broad and overlapping character of O-H bands, these features should be treated as indicators of modified hydrogen bonding rather than as direct evidence of a single type of interaction.

**Figure 4:**
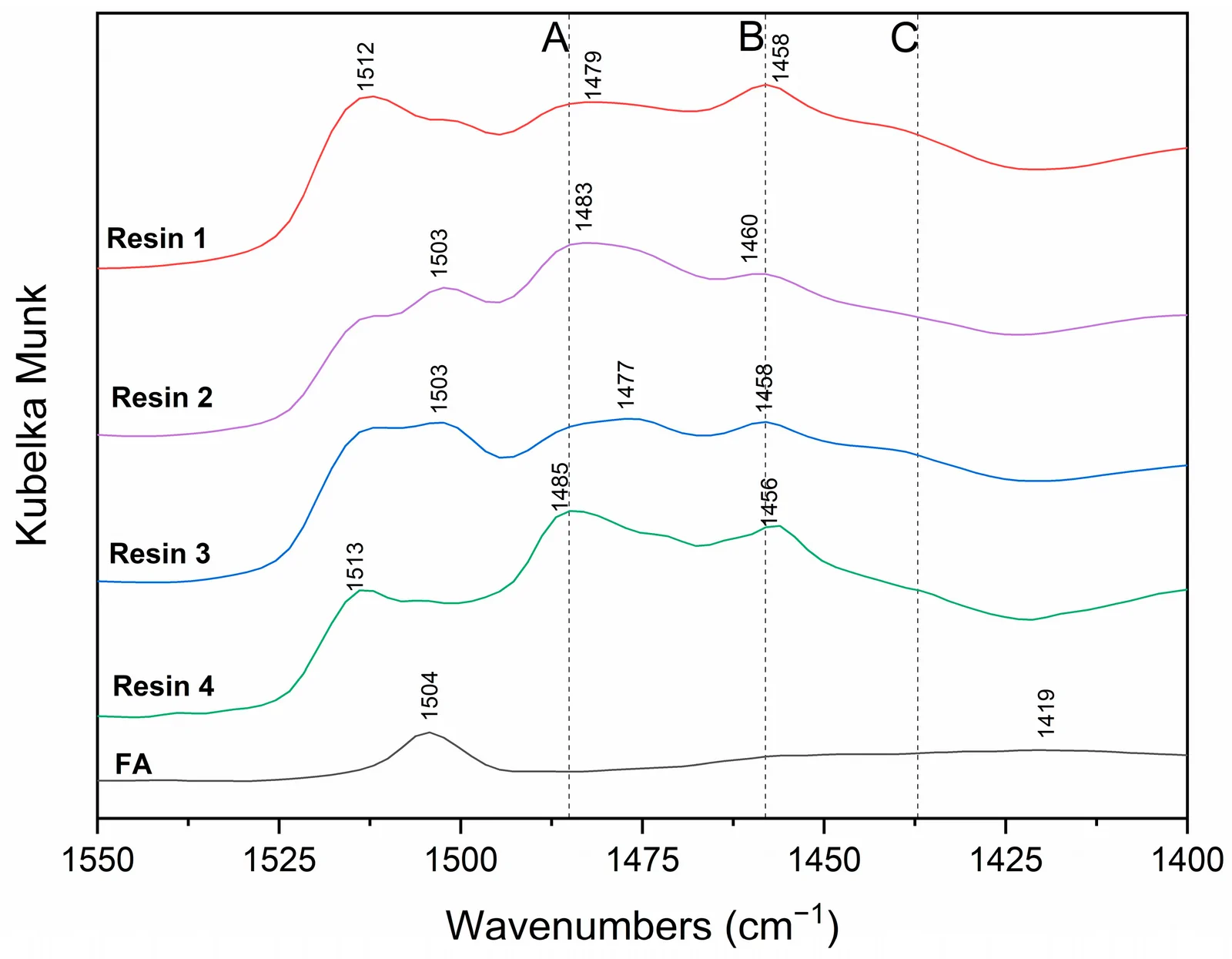
Room temperature DRS spectra of studied Resins 1–4 and FA in the range of methylene bridges.

The bands at 3020 cm^−1^ and 2900 cm^−1^ come from C-H vibrations in the phenol ring and formaldehyde (stretching in aromatic methoxyl groups/aliphatic methyl and methylene groups of side chains and stretching in aromatic methoxyl groups and methyl/methylene groups of side chains). Two intense bands at 1610 and 1597 cm^−1^ can be attributed to vibrations in the phenol ring. The first one is probably the result of the appearance of 4-hydroxybenzaldehyde, a product of the initial reaction of phenol with formaldehyde (an intermediate product in the resin hardening process). Both bands originate from C=C stretching vibrations in the phenyl ring. In the samples containing FA (Resin 2 and Resin 3), the first of the aforementioned bands has a lower intensity than in the samples without FA (Resin 1 and Resin 4). This doublet of C=C bands can probably be considered an indicator of the presence of methylol groups in the phenyl ring at positions 1,2 and 1,4. The first of the aforementioned positions will be responsible for ortho connections, while the second for para. Based on FT-IR observations it is likely that FA “favors” the para configuration, as the intensity of the para-attributed bands in FA-modified resins is increased at the expense of ortho-attributed ones. In contrast, resins without FA have almost equal intensities of the two bands. Nevertheless, the observed doublet evolution should be interpreted as a comparative spectral trend rather than a direct quantitative measure of ortho and para substitution. The hydroxymethyl vibration region at approximately 1000 cm^−1^ also changes after the addition of FA. In its absence, this band has two components at 996 and 1020 cm^−1^. The former can again be attributed to the ortho positions and the latter to the para. The addition of FA shifts the position of the 1020 cm^−1^ band to 1010 cm^−1^ and significantly reduces the intensity of the one at 996 cm^−1^. Again, this suggests favoring the para position. Additional information is provided by the aromatic bands at approximately 760 and 820 cm^−1^. They are responsible for C-H out-of-plane bending in aromatic rings. Bands in this range are commonly sensitive to the substitution of aromatic rings. The former can be attributed to vibrations at the 1,2-substitution, while the latter can be attributed to vibrations at 1,4. For both types of resin, the tendency is the same—the addition of FA changes the relative intensities of these bands, with an increase in para bonds. This supports the interpretation that FA influences the condensation pathway. The literature states that the most important bands from the point of view of the resin cross-linking are in the range of 1550–1400 cm^−1^ [19], as shown in Figure 4.

**Figure 5:**
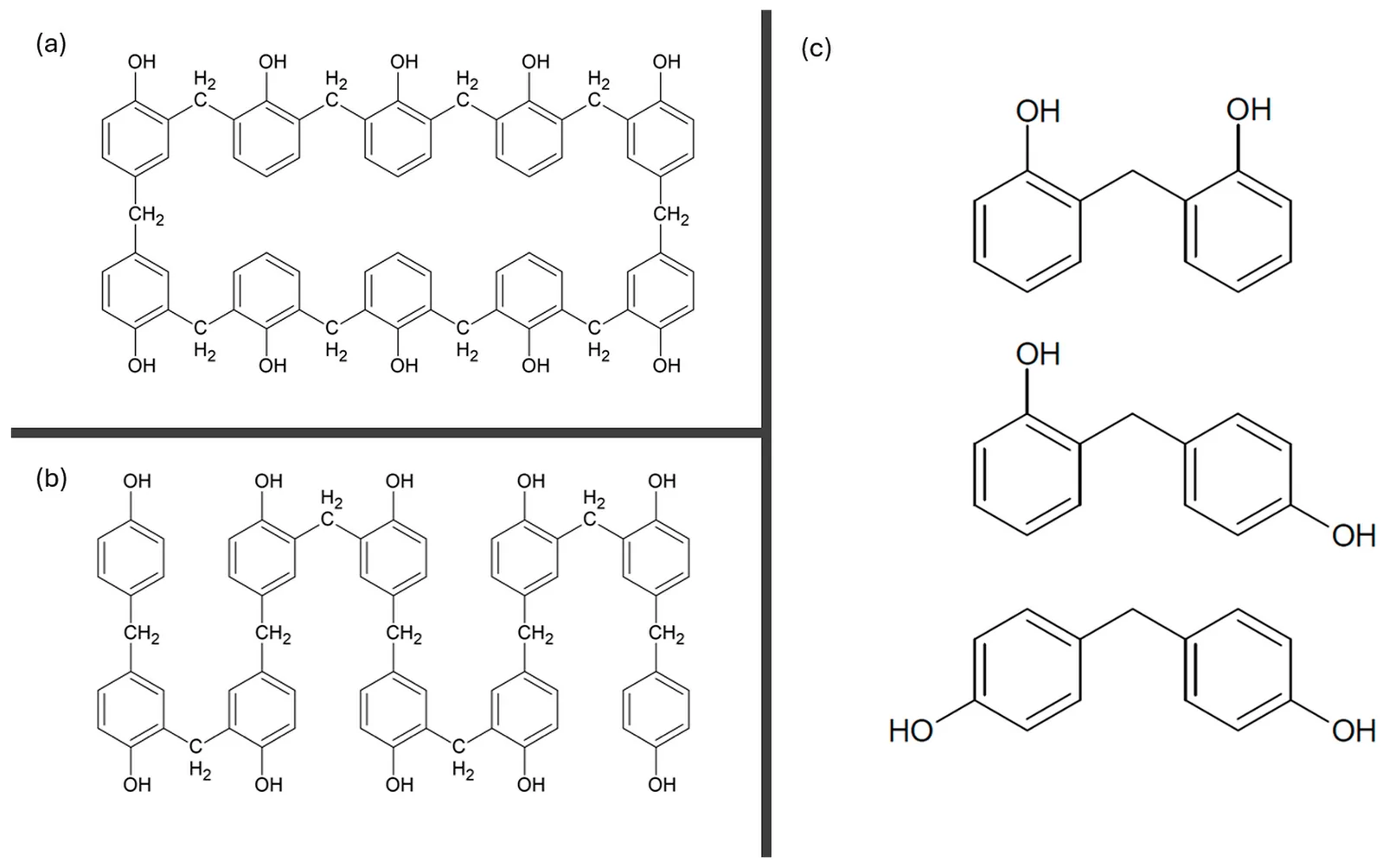
Phenol-formaldehyde resin with a predominant share of: (**a**) o-o bonds (**b**) p-p bonds. (**c**) Three different types of connections between phenol rings o-o, o-p, p-p.

The presented comparison shows that each of the phenolic resin samples contains bands at approximately 1512 cm^−1^, which originate from C=C vibrations in the phenyl ring. The 1503 cm^−1^ band, characteristic of C=C stretching in FA, is also visible, with higher intensities observed for the FA-modified samples. The bands at approximately 1480, 1460 and 1440 cm^−1^ come from methylene bridges connecting phenol rings and these are the most characteristic bands that can be used to analyze the cross-linking process. The first belongs to p-p connections (A), the second to ortho-para (o-p) (B) and the third belongs to o-o connections (C)—Figure 4 and Figure 5c [23]. These bands are strongly overlapped and their apparent intensities may be affected by the neighboring aromatic and FA-related vibrations. For this reason, they are not treated here as fully separated quantitative markers of individual bridge populations. Instead, the evolution of this spectral envelope is used as a comparative indicator of changes in the methylene bridge environment.

Comparison of the FA-free and FA-containing resin pairs shows that the addition of alcohol modifies the shape of the 1550–1400 cm^−1^ spectra. In the Resin 1 vs Resin 3 pair, the dominant contribution shifts from the region near 1460 cm^−1^ toward the 1480 cm^−1^ wavenumber after FA addition. A similar tendency is observed in the Resin 4 vs. Resin 2 pair, where the band near 1480 cm^−1^ becomes more pronounced in the FA-containing sample. At the same time, the band near 1440 cm^−1^ remains relatively weak in all studied resins. These observations suggest that the samples containing alcohol exhibit spectral features consistent with a higher relative contribution of para-associated methylene structures Figure 5a,b. It can be assumed that the reaction in the para position is thermodynamically favored. This can be explained by the formation of polymer networks with a higher proportion of linear segments, which can arrange themselves parallel to one another. In such an arrangement, van der Waals interactions may form, as well as π-π stacking of aromatic rings is also possible.

### 3.2. FT-IR Temperature-Resolved Analysis

**Figure 6:**
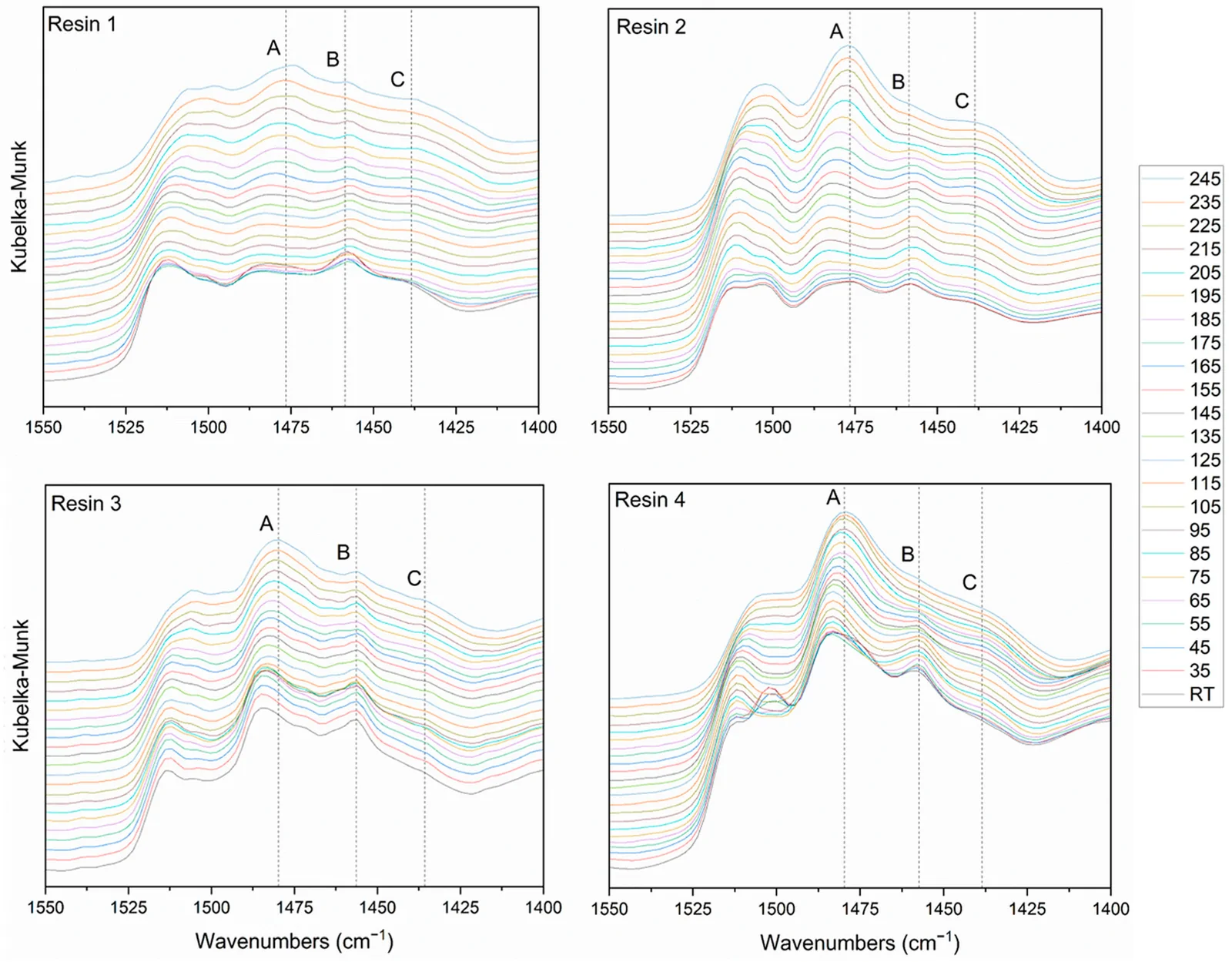
DRIFTS spectra of selected Resins 1–4.

Figure 6 shows the spectra of phenolic resins as a function of temperature. Resins 1 (F110 commercial and Resin 4 (MC-05 without FA) lack FA while Resin 2 (MC-05 commercial) and Resin 3 (F110 with FA) are FA-modified.

The temperature range up to 250 °C was selected as an analytical post-curing window rather than as a recommended technological curing temperature. Although the main curing of PF resins typically occurs at lower temperatures, late-stage condensation, hydroxymethyl consumption, rearrangement of methylene/ether-related structures and volatile release may continue at higher temperatures. Thus, the extended temperature range was used to follow completeness and the direction of structural evolution in the investigated systems.

In FA-absent Resin 1, the low-temperature spectrum shows a relatively broad spectral envelope with noticeable contributions in the region associated with both ortho- and para- methylene structures (Figure 6). Upon heating, above approximately 150 °C, the spectra gradually shift toward a stronger relative contribution of p-p bonds (A). In Resin 3, the FA-containing counterpart of Resin 1, this contribution is already more visible at lower temperatures and remains pronounced during heating. This suggests that FA modifies the initial structure of the resin and promotes a curing pathway in which p-p spectral features are relatively enhanced.

A similar tendency is observed for the Resin 4/Resin 2 pair. Resin 4, which does not contain FA, exhibits a more balanced contribution of the 1480 (A) and 1460 cm^−1^ (B) regions at room temperature (p-p vs. o-p). In contrast, Resin 2, which contains FA, shows a more pronounced contribution of the region near 1480 cm^−1^ throughout the investigated temperature range. This supports the interpretation that samples containing FA exhibit spectral changes consistent with a higher relative contribution of p-p methylene bridges. Overall, the temperature DRIFTS results indicate that FA affects both the initial spectral state of PF resins and their evolution during heating. Since p-p connections are opposite to one another and have the lowest steric hindrance, they are the most energetically favored. This is followed by o-p, with o-o (1440 cm^−1^-C) being the least favored. Yet, because there are twice as many ortho sites in phenol, in the absence of any additives, the ratios of p-p, o-p and o-o should be equal. While Resin 1 had similar ratios of all the configurations (with small temperature-induced alterations) in Resin 2, p-p was always favored. It should also be noted that Resin 2 and Resin 4 belong to a different commercial composition family than Resin 1 and Resin 3 with a different catalyst, which can also alter the starting p-p, o-p and o-o ratios. All in all, these results suggest partial steric hindrance of para positions in Resin 1 and ortho in Resin 2.

In both FA-containing resins (Resin 2 and 3), the addition of alcohol shifted the balance towards formation of p-p bridges and this effect became more pronounced with increasing temperature.

**Figure 7:**
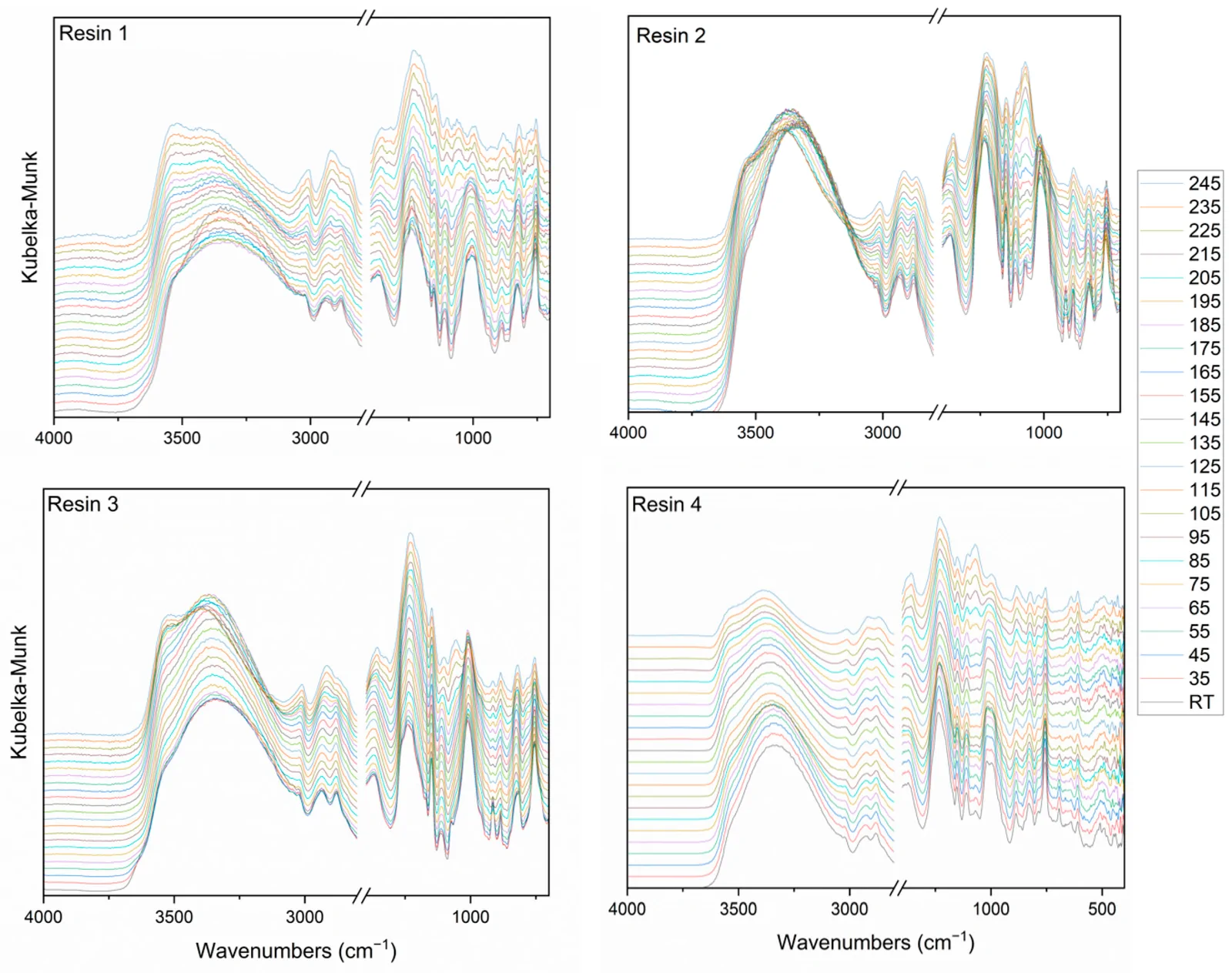
Temperature-resolved DRIFTS spectra of the investigated resins acquired during continuous heating from room temperature to 245 °C: Resin 1 (FA-free commercial F110), Resin 2 (FA-containing commercial MC-05), Resin 3 (FA-modified F110) and Resin 4 (FA-free MC-05).

As shown in Figure 7, Resin 2 and Resin 4 exhibit relatively similar spectral features at room temperature in the methylene bridge region, despite the presence of FA in only Resin 2. This suggests that the initial spectral envelope is not only influenced by the addition of FA, but also by the composition of the resin, including the catalyst system. Therefore, similarities between the selected systems, both with and without FA, should not be considered mechanistically identical. FA most likely modifies the curing process through hydrogen bonding and the participation of furan-derived structures, whereas Ba^2+^ may influence the curing pathway through cation coordination, ion-pairing with phenolate species and changes in the local organization of hydroxyl-containing groups [24,25].

DRIFTS results suggest that FA may interact with phenolic and hydroxymethyl groups through hydrogen bonding and may modify the accessibility (steric hindrance) or reactivity of para and ortho sites, promoting p-p bridge formation. This tendency became more pronounced as the temperature increased. This seems to reduce the reaction kinetics, but makes them more stable, yielding products with higher crosslinking efficiency. This result is in accordance with our previous study, where an improvement in the quality of carbon fiber composites was achieved by using a resin modified with furfuryl alcohol [22].

Comparisons within the Resin 1 vs. Resin 3 and Resin 4 vs. Resin 2 pairs are the most suitable for discussing the effect of FA, whereas differences between the two resin families may additionally reflect composition and catalyst-related effects. Figure 7 presents the temperature-resolved DRIFTS spectra of all four investigated resins. In the O-H stretching region, all samples show temperature-dependent changes, but their extent differs among the resin systems. In the FA-free resins, especially Resin 1, the broad O-H band (centered near 3350 cm^−1^) more rapidly decreases in intensity with increasing temperature, which is consistent with progressive condensation and the consumption of hydroxyl-containing groups. In the corresponding FA-containing sample (Resin 3), this decrease is less pronounced, suggesting that FA modifies the hydrogen bonding environment and broadens the temperature where hydroxyl species remain involved in the curing process. A similar trend is observed for the Resin 4/Resin 2 pair, where the FA-containing Resin 2 also shows a more gradual evolution of the O-H region compared with FA-free Resin 4. We can also observe differences in the range of C=C vibrations in the aromatic rings (bands at approximately 1600 cm^−1^). For the alcohol-free samples, the doublet disappears and a single band appears at approximately 1600 cm^−1^, while for the samples with FA (Resin 2 and Resin 3), there is an inversion of the doublet intensity ratios, which may also indicate a preferred direction of the reaction—towards p-p connections as these are only visible in the FA-containing samples. This is manifested by an increased intensity of the band at 1597 cm^−1^ in Resins 3 and 2 (with FA), as compared to Resin 1 and 4 (without FA).

In the case of both materials, we observe the disappearance of the band at approximately 1010 cm^−1^, which may be an indicator of the course of the cross-linking reaction with methylene bridges of phenolic resins. When the bonds are saturated, this band disappears, which occurs at 190 °C in FA-absent materials, but does not happen before 250 °C in FA-modified ones. This may, therefore, indicate that FA affects the temperature range required to cross-link a given resin.

Overall, the DRIFTS comparison of Resin 1 and Resin 3 supports the hypothesis that FA moderates the curing process and promotes a more gradual structural evolution of the PF network. At the same time, the comparison of the two resin families indicates that the detailed spectral evolution is also influenced by the initial resin composition as well as the catalyst used.

### 3.3. ^1^H and ^13^C MAS-NMR

To provide complementary structural information to the DRIFTS analysis, solid-state ^1^H and ^13^C MAS-NMR spectra of the investigated resins were recorded.

**Figure 8:**
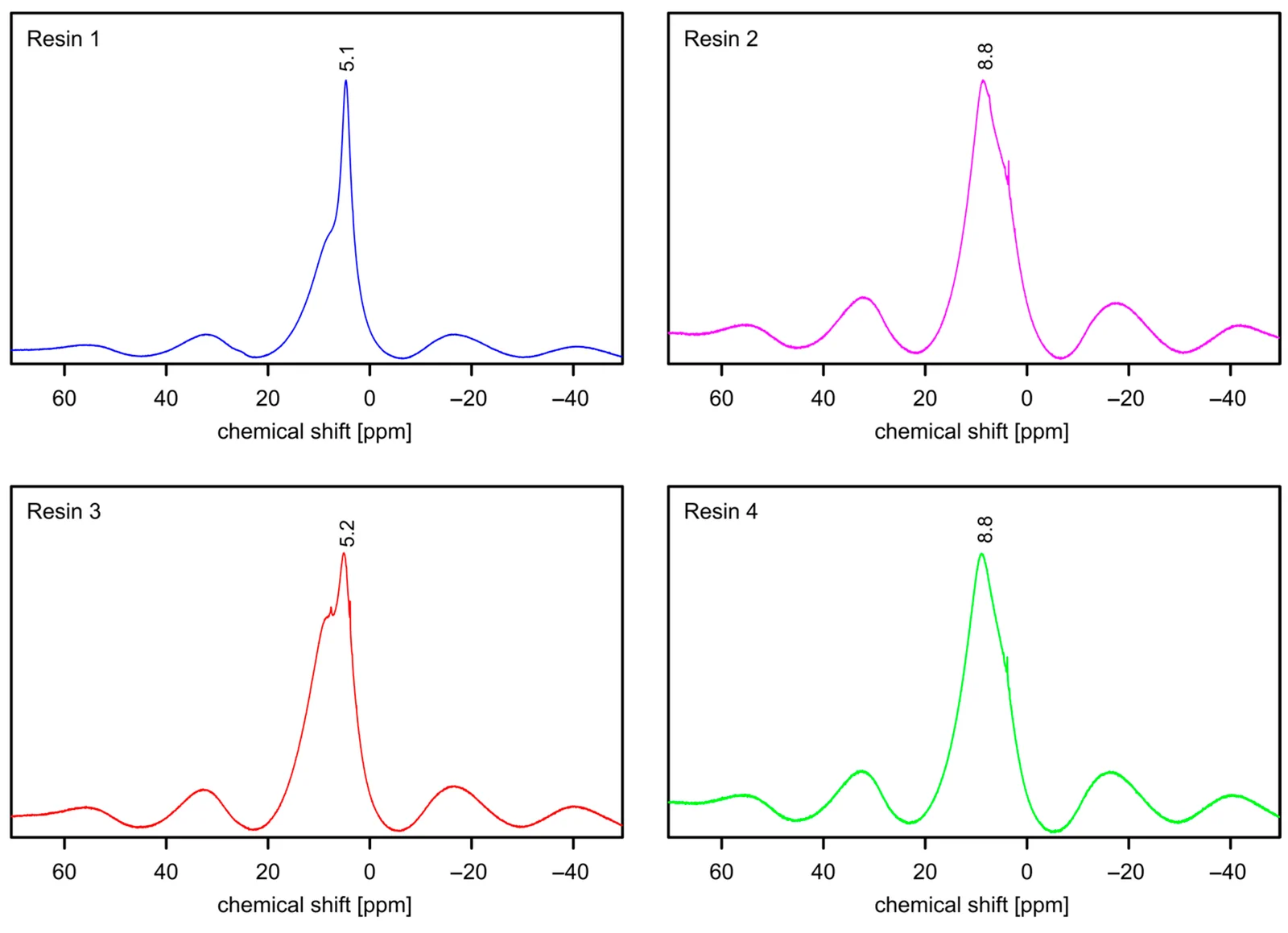
^1^H MAS-NMR spectra of investigated resins: Resin 1 (FA-free commercial F110), Resin 2 (FA-containing commercial MC-05), Resin 3 (FA-modified F110) and Resin 4 (FA-free MC-05).

The ^1^H MAS-NMR spectra of the investigated resins are presented in Figure 8. Due to the rigid and heterogeneous character of cured PF networks, the spectra exhibit broad resonances accompanied by spinning sidebands typical of solid-state MAS-NMR measurements [26]. Therefore, the discussion is focused mainly on the dominant isotropic resonances rather than on individual sideband features.

A clear difference is observed between the two resin systems. The F110 samples, Resins 1 and 3, show the dominant resonance at approximately 5.1–5.2 ppm, while the MC-05 samples, Resins 2 and 4, exhibit the main resonance at approximately 8.8 ppm. The similarity of the ^1^H MAS-NMR spectra within each resin system, despite the presence of FA only in Resin 2 and Resin 3, indicates that the overall proton environment is largely governed by the initial resin composition [26]. This observation supports the conclusion that the spectral differences between the F110 and MC-05 systems cannot be interpreted solely as the effect of FA addition. The shift in the main ^1^H resonance toward higher chemical shift values in the MC-05 resins suggests a different local proton environment, possibly related to stronger hydrogen bonding, a different phenolic/hydroxymethyl arrangement or changes in the organization of hydroxyl groups. This may be caused by the starting composition and the catalyst used. In particular, the presence of Ba^2+^ in the MC-05 resins may influence the local arrangement of oxygen-containing groups through cation coordination and/or ion-pairing with phenolate species. However, this effect should not be treated as mechanistically identical to the role of FA. FA may modify the curing process through hydrogen bonding and the contribution of furan structures, whereas the catalyst system may affect the local organization and reactivity of hydroxyl and phenolate groups.

**Figure 9:**
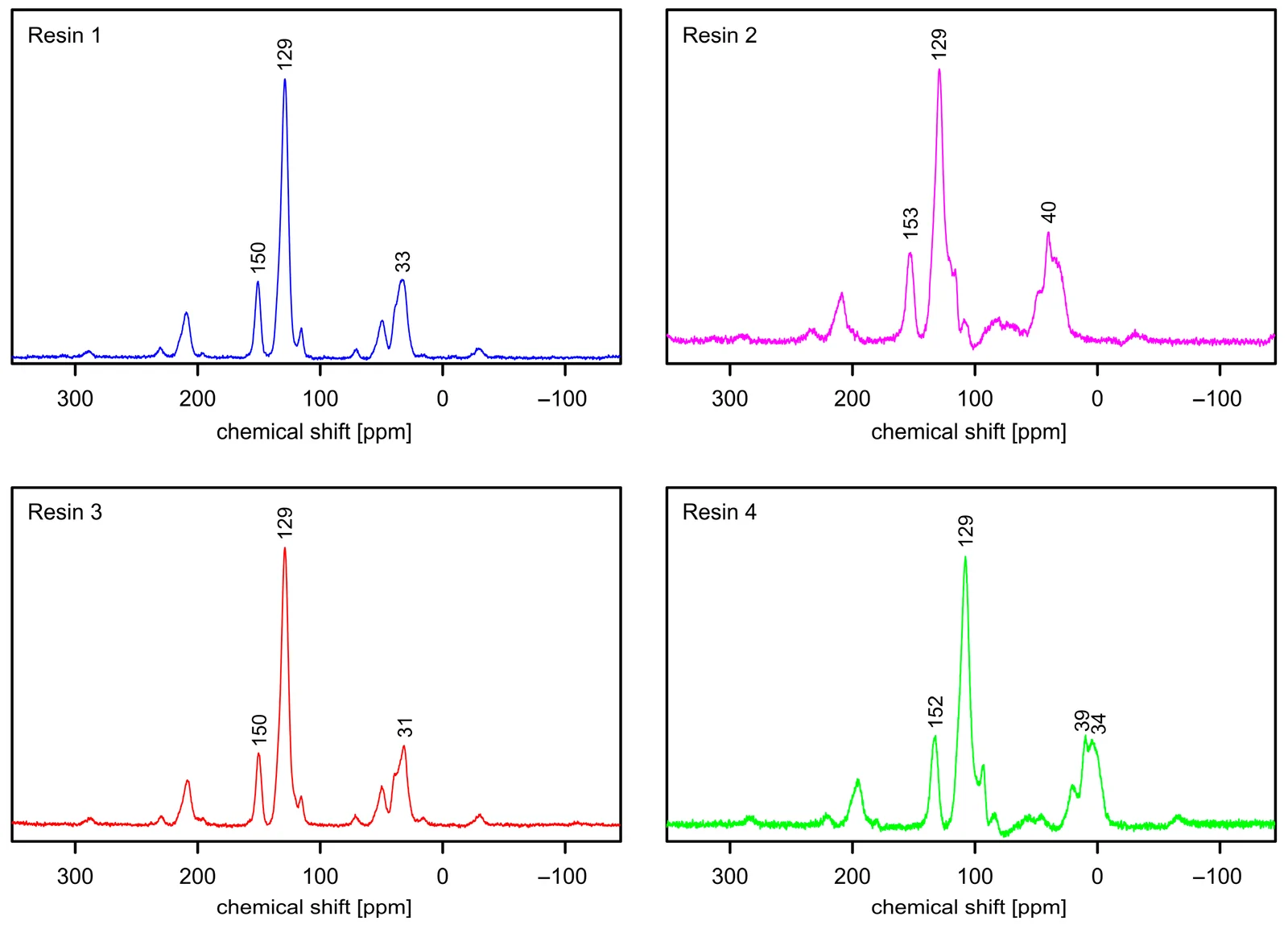
^13^C MAS-NMR spectra of investigated resins: Resin 1 (FA-free commercial F110), Resin 2 (FA-containing commercial MC-05), Resin 3 (FA-modified F110) and Resin 4 (FA-free MC-05).

All ^13^C NMR-tested samples (Figure 9) exhibit intense resonances in the aromatic region, with the dominant signal located at approximately 129 ppm [27,28]. This signal can be assigned mainly to aromatic carbon atoms of phenolic rings, with possible overlap from furan and/or derivative carbon environments in FA-containing systems. Signals located at approximately 150–153 ppm correspond to oxygen-substituted aromatic carbon atoms, e.g., phenolic C-O environments and may also contain contributions from carbon atoms in FA-modified samples [27,28]. The aliphatic region between approximately 31 and 40 ppm is particularly relevant for discussing the curing process. According to literature data for resol PF resins, resonances at 39–42 ppm are commonly associated with p-p methylene bridges, whereas those at 34–36 ppm and 29–31 ppm are attributed to p-o and o-o methylene bridges, respectively [27,28,29,30]. In the present solid-state MAS-NMR spectra, these assignments are used as qualitative indicators of methylene bridge environments, rather than as quantitative measures of individual bridge populations, because the spectra are broadened and may contain overlapping contributions from different local environments.

Resin 1 and Resin 3, representing the FA-free and FA-modified F110 systems, show similar aromatic carbon environments, while differences are observed in the methylene region. This indicates that FA does not completely change the aromatic backbone of the resin, but modifies the local aliphatic bridge environment involved in the network formation. The MC-05 resins (Resin 2 and 4) also show similar main aromatic resonances. This observation supports the conclusion drawn from DRIFTS that the initial structure of the resin cannot be interpreted solely in terms of FA addition. Composition-related factors, including the catalyst used, may also influence the local organization of phenolic, hydroxymethyl and methylene bridge environments. In particular, the more pronounced signals in the 34–40 ppm region observed for the MC-05 systems suggest differences in the methylene bridge environments, as compared with the F110 resins.

### 3.4. XPS

**Figure 10:**
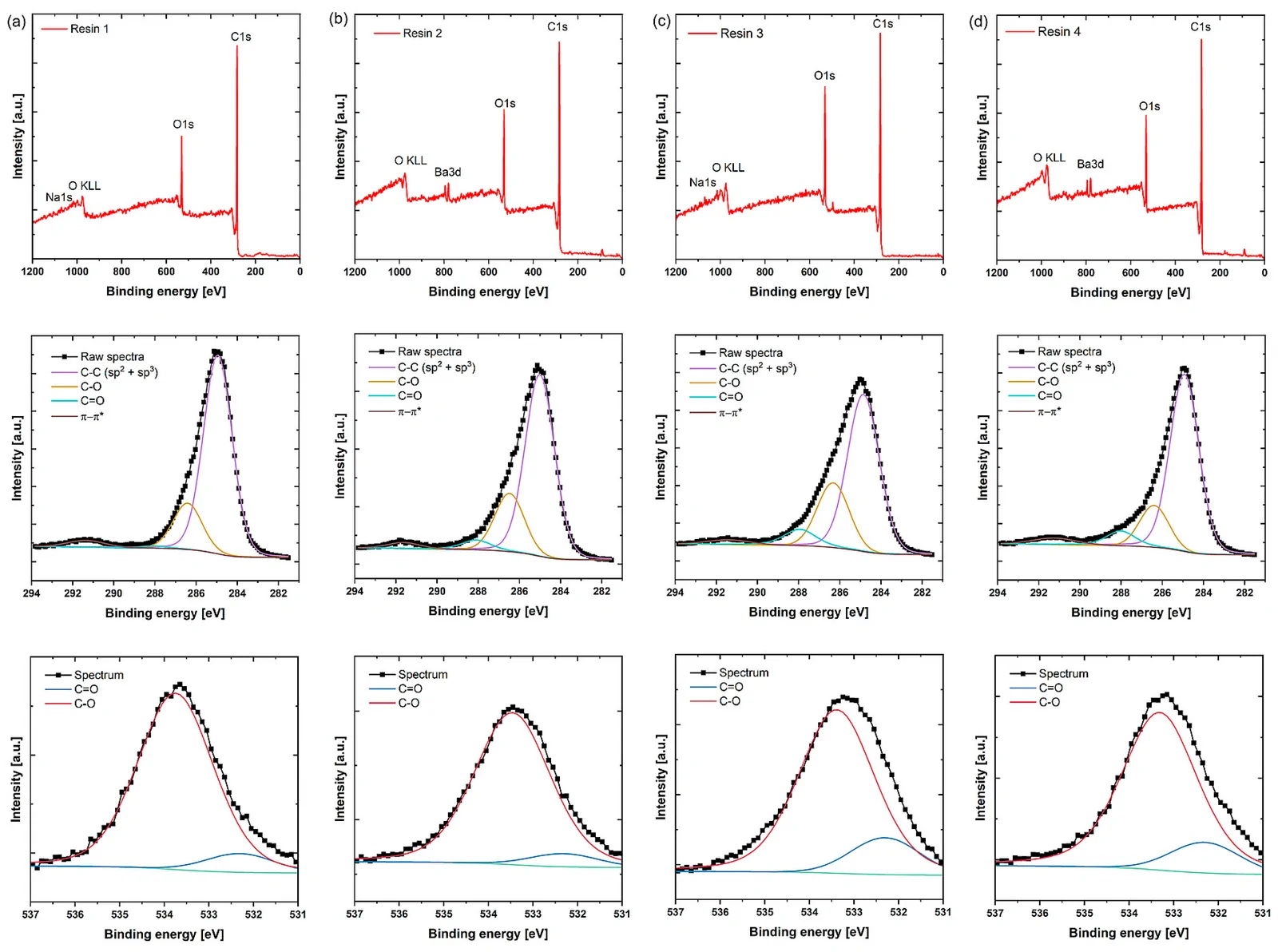
XPS survey spectra, C1s and O1s core level spectra for (**a**) Resin 1 (**b**) Resin 2 (**c**) Resin 3 (**d**) Resin 4.

The chemical composition of the surface of the cross-linked resins without FA (Resin 1 and 4) and with FA (Resin 2 and 3) with different basic catalysts (Na and Ba) was investigated using XPS and the recorded spectra are shown in Figure 10. The survey scan revealed the presence of lines corresponding to carbon, oxygen, sodium, barium and related Auger transitions. The valence states of present elements were resolved by fitting high-resolution core-level spectra, done to gain insight into the chemical structure of the cured resins. The C1s line was fitted with four components attributed to C-C/C-H/C=C (ar.) (sp^2^ and sp^3^) bonds, originating from aromatic phenolic rings and methylene bridges at 285.0 eV; C-OH/C-O-C, hydroxylic and/or ether bonds at 286.4 eV; C=O carbonyl-like species and O-C-O structures formed during condensation and furan-related reactions at 287.9 eV; and a weak signal observed at 291.4 eV attributed to a π-π* aromatic carbon shake-up transition (Table 3) [31,32,33,34]. The O1s region revealed two components located at 532.3 eV corresponding to oxygen double-bonded with carbon O=C and at 533.3 eV corresponding to single-bonded oxygen with carbon and other elements (O-C, O-H, O-Na, O-Ba) [32,33]. The lines from sodium and barium had 1072.4 and 779.9 eV peaks, respectively [33,35] attributed to the basic synthesis agents used: NaOH (in Resins 1 and 3) and Ba(OH)_2_ (in Resins 2 and 4) at content varying from 0.2 to 0.8%. The analysis of samples differing by the FA presence and the alkali agent types, allowed us to ascertain a distinct influence of FA on the curing kinetics of the investigated resins.

Resin 1 exhibited the highest concentrations of C-C/C-H species (66.9%) and the lowest content of oxygen-containing groups, indicating a surface dominated by the phenol-formaldehyde network. In this NaOH-catalyzed system, the comparison of Resin 1 and Resin 3 clearly shows that the incorporation of FA led to a pronounced decrease in the C-C component (from 66.9% to 52.7%), accompanied by an increase in C-OH/C-O-C groups from 14.5% to 20.9%, C=O groups (from 0.8% to 4.9%), and O1s oxygen-containing species. These results are consistent with the successful incorporation of furfuryl alcohol into the resin structure and indicate that FA addition significantly increased the amount of oxygen-containing surface functionalities.

A similar, although less pronounced, tendency was observed for the Ba(OH)_2_-catalyzed system. In comparison with Resin 4, Resin 2 showed a lower content of C-C/C-H bonds (60.4% vs. 63.5%) and a markedly higher contribution of C1s component C-OH/C-O-C groups (19.1% vs. 13.7%). However, the carbonyl components were not significantly increased in Resin 2 relative to Resin 4, suggesting that in this catalytic system the influence of FA is mainly reflected by the higher amount of singly bonded oxygen species rather than in the formation of additional carbonyl functionalities.

The increased content of single-bonded oxygen is related to the presence of C-O-C bonds in furfuryl rings and possibly to the slower cross-linking kinetics, resulting in a higher number of C-OH groups from unreacted methylol phenol and furfuryl alcohol. This increase in the -OH groups in Resin 2 and Resin 3 after curing was also confirmed by the DRIFTS spectra presented above. The hypothesis of slower polycondensation kinetics is supported by our previous findings, which showed a reduced water release from Resin 2, as evidenced by mass spectrometry (MS) of the evolved gases during cross-linking [22]. Alternatively, the higher amount of C-O bonds could be attributed to surface-confined furfuryl alcohol or water. However, based on the reasoning above, such an explanation appears unlikely.

### 3.5. STA—FT-IR and Rheological Analysis

**Figure 11:**
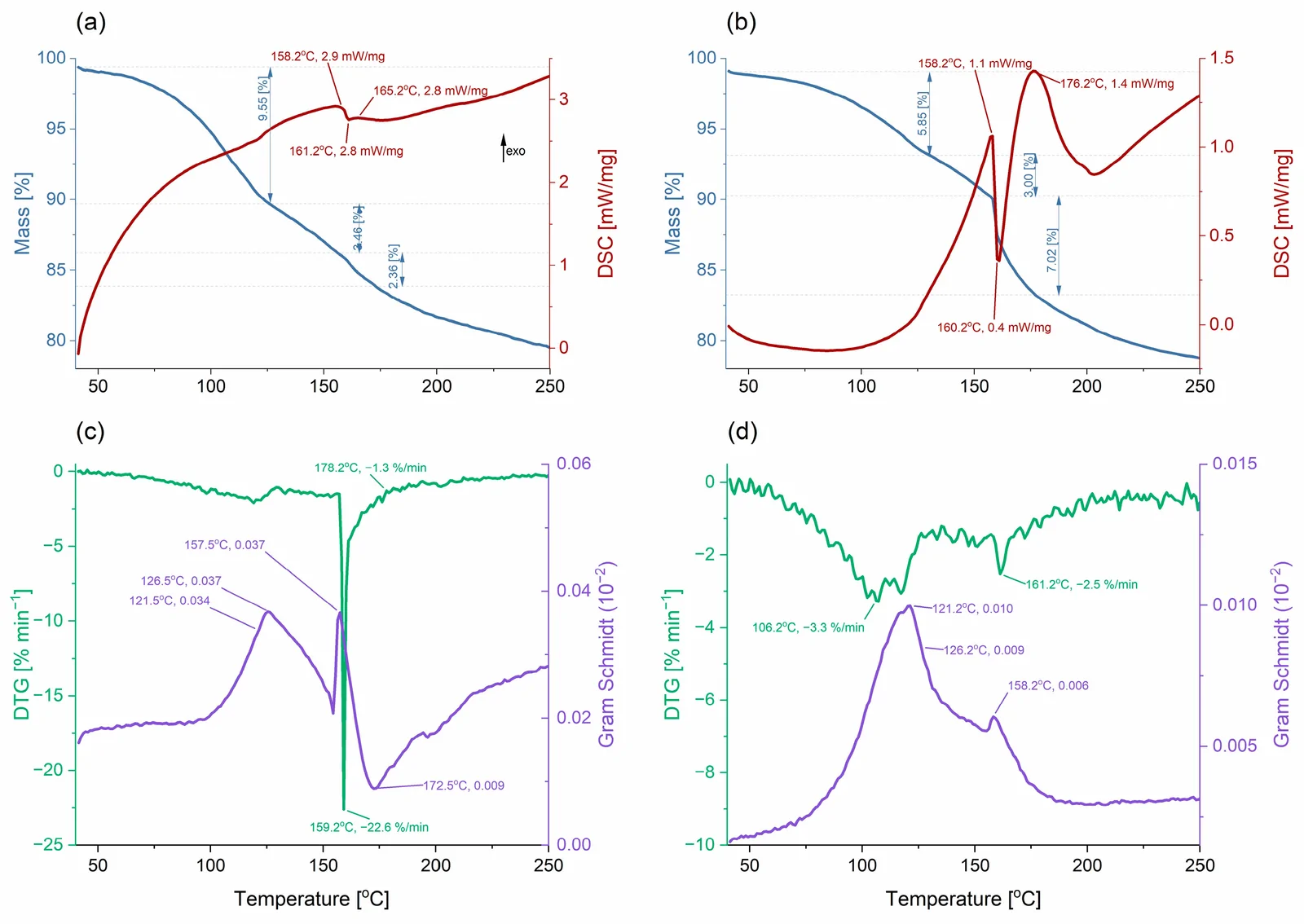
TGA and DSC curves for cross-linking process of commercial Resin 1 (**a**) and Resin 2 (**b**), presented together with corresponding Gram-Schmidt and DTG curves of Resin 1 (**c**) and Resin 2 (**d**).

STA-FT-IR analysis was conducted to confirm the observations obtained through DRS and DRIFTS analyses. The TGA, DSC, (derivative thermogravimetry) DTG and Gram-Schmidt (GS) curves, presented as a function of increasing temperature, are shown in Figure 11. A GS curve is a mathematical reconstruction of the overall change in the infrared adsorption registered by the FT-IR detector. It is obtained using the GS algorithm, which calculates the total infrared absorbance of evolved gases by operating directly on raw time-domain interferograms, bypassing the Fourier transform. Initially, baseline interferograms of the empty purge gas are used to construct an orthogonal set of basis vectors, defining a background vector space. As the sample thermally degrades, each new interferogram is treated as a vector and projected onto this background space. The algorithm subtracts this background projection from the raw signal to isolate a residual vector. The scalar magnitude of this residual vector yields the GS intensity, which is directly proportional to the total concentration of all IR-absorbing gases released at that specific time or temperature. Hence, GS curves can be used to monitor the overall evolution of gaseous products [23,36].

Additional analyzed curves can be found in the Supplementary Materials (Figures S1–S5). 3D STA-FT-IR plots for Resin 1 and 2, the results of the EPA/NIST library search of the spectra recorded at the temperature of maximum release of certain spectral bands and gas evolution curves for specific volatiles are presented in the Supplementary Materials (Figures S6–S13).

A summary of the STA-FT-IR results can be found in Table S1. Analyzing the obtained curves, one can see stark differences in their trends. In the absence of FA (Resin 1) and up to 159 °C, mass loss proceeds slowly and steadily, at a maximum rate slightly exceeding 2% min^−1^ (Figure S1). Total mass lost up to this stage equals 8.7%. Gaseous products start to evolve around 87 °C, reaching a maximum at 126 °C. Analyzing the FT-IR spectra, one can see that in this range, the products released are mostly methane, some phenol, supplemented with a small amount of water. The reactions occurring are exothermic—most likely, crosslinking. At 158 °C, the onset of an overlapping endothermic reaction is observed, which peaks at 161 °C. and ends at 178 °C. The amount of heat consumed is 33 J g^−1^. This is accompanied by a significant drop in mass, which peaks at 23% min^−1^ at 159 °C. The total mass loss equals to 7%. At the same time, the second maximum of volatile release is registered, with increasing amounts of phenol, methane and water. Upon completion of the exothermic reaction, co-occurring endothermic reactions become more evident. These end at around 206 °C, accompanied by a mass loss of 2.6% and a release of phenol with CO. Residual mass at 250 °C is equal to 78.8%. In Resin 1, exothermic crosslinking is effectively catalyzed by temperatures exceeding 87 °C (most effectively, above 122 °C), but competitive thermal decomposition, leading to the production of methane and phenol, can undermine effective and complete crosslinking.

Completely different kinetics of mass loss and energetic reactions are observed in Resin 2, which contained FA in its commercial variant. Here, the mass loss rate is mostly monotonic, with two small maxima at 107 and 161 °C, reaching 3.3 and 2.5 °C min^−1^, respectively. Despite the presence of similarly overlapping endothermic and exothermic transitions (the latter peaks at 161 °C), the magnitude of the effects is markedly lower than that observed for Resin 1. There is almost monotonic heat consumption observed from the beginning (as early as 40 °C), indicating stable, undisturbed crosslinking. Volatile production is generally low, starting at around 87 °C and ending at 192 °C, with two maxima at 122 and 169 °C. Similarly to Resin 1, the second maxima of gas release corresponds to the exothermic effect observed in the DSC curve. In this resin, above 150 °C a small increase in water release is observed and the effect observed in DSC is not correlated with any other volatile compound, suggesting no thermal decomposition takes place. All in all, in Resin 2, there is a small amount of phenol, methane and FA released from around 75 to 175 °C, with a peak at around 114 °C. There is also a stable, almost monotonic release of water and CO_2_. These results suggest stable crosslinking, with no thermal decomposition.

Comparing the STA-FT-IR data with the DRIFTS spectra, it becomes apparent that there are significant differences in the water-attributed bands. There is a significant amount of water in a liquid state observed in DRIFTS—i.e., at the surface of materials—but the amount of water vapor that reaches the gaseous detector in STA-FT-IR is rather low. This might indicate that water is somehow consumed at the surface of the sample and does not evaporate. It is possible that it undergoes some reactions with other volatile products or the components of the resin. One example of such a reaction could be the hydrogenolysis of furfuryl alcohol [37]. This typically requires a catalyst, which might have been present in the commercial resin. This could result in minor side reactions with the hydroxymethyl groups or other reactive species present in the mixture. Water itself could also induce partial hydrolysis of the resin or its by-products. It is also possible that the nitrogen flow used does not allow the reaction by-products to reconstitute fully into water.

When comparing the results of the FT-IR analysis of gaseous products with those obtained using MS in our previous studies [22], differences in the main constituents also become apparent. While in MS water was the main gaseous compound in resins (both with and without FA), FT-IR found that the main compound for Resins without FA is methane and phenol and for resins with FA it is carbon dioxide + FA. We repeated the measurements several times and always found water to be residual in STA-FT-IR. A possible explanation for the differences between MS and FT-IR comes from the different experimental setups of the two. In MS, the samples were measured in aluminum crucibles, under a protective flow of helium and the measurements started at 25 °C. In the FT-IR setup, the samples were placed in ceramic crucibles which can physisorb some of the water. The samples were heated to 40 °C and held at that temperature for 10 min before the measurements started and the FT-IR detector started recording the actual sample. Immediately prior to that, the device measures a background signal, which is a signal that reaches the detector before the heating starts. It is possible that during this initial heating and stabilization step, water was steadily evaporating from the sample and reaching the detector. If the levels were higher than those of the water released during the actual heating, the signal would be completely removed from the sample spectra. Importantly, while the amounts of water released are small in both samples, there are still some differences observed. In Resin 2, which contained FA, a stable release was recorded, indicating crosslinking in progress. In Resin 1, 2 peaks of maximum release were recorded between 100 and 150 °C—roughly mirroring the methane and phenol release. This indicates that in Resin 1, water release might be correlated with the thermal decomposition. The observed differences in reaction kinetics may also be attributed, at least in part, to the different alkaline curing agents used in the investigated resins.

**Figure 12:**
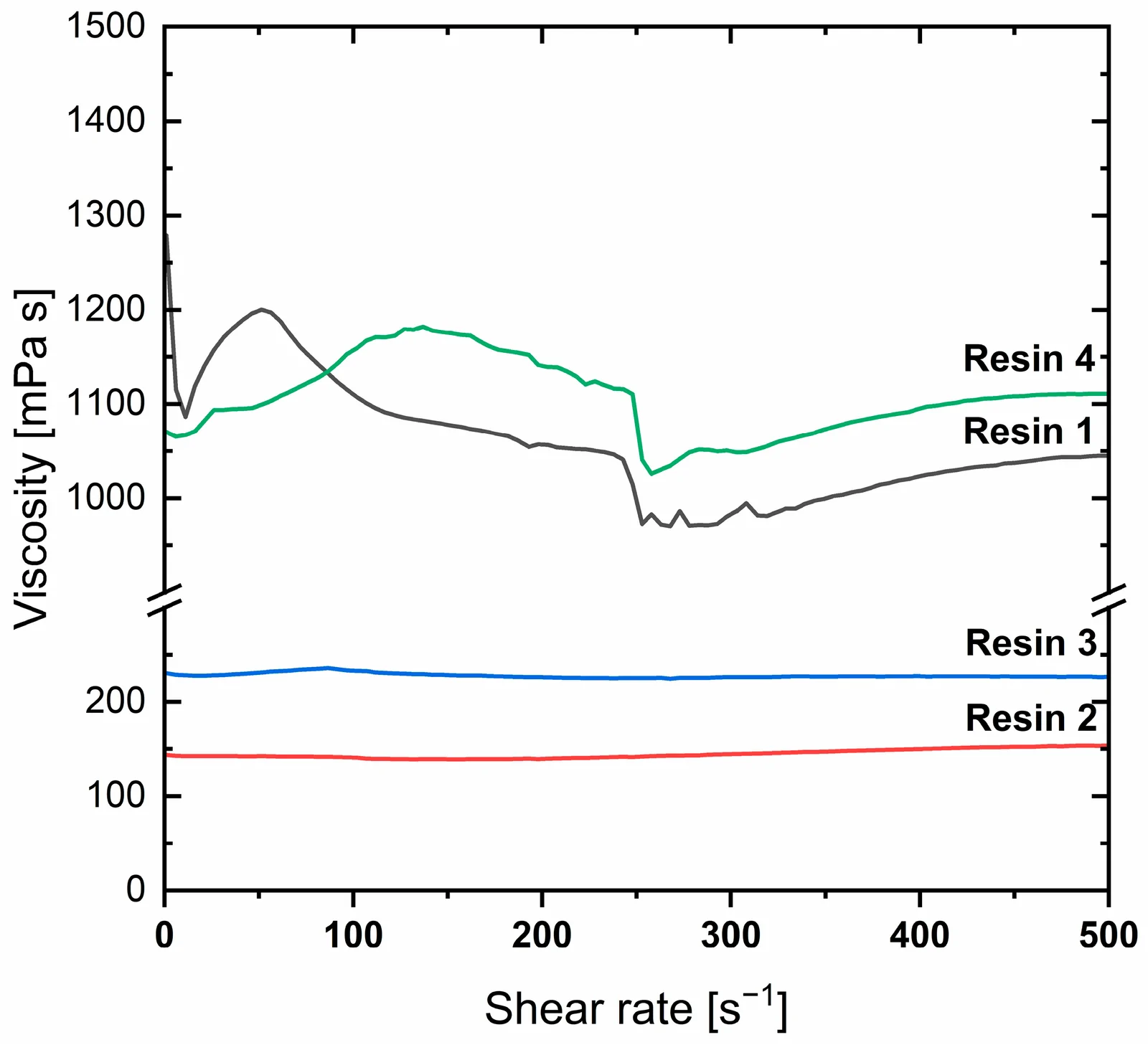
Dynamic viscosity as a function of shear rate at 30 °C for Resins 1–4.

In addition, the dynamic viscosity of the investigated resins was measured to assess their suitability for composite manufacturing processes [38,39] (Figure 12). At 30 °C, the dynamic viscosity was 1051 mPa s for Resin 1 and 144 mPa s for Resin 2. The influence of FA on viscosity reduction was further confirmed by measurements performed on Resin 3 and Resin 4, which exhibited viscosities of 228 mPa s and 1104 mPa s, respectively.

When considered together with the findings of our previous study [22], which indicated that FA prolongs the gelation time of PF resins and contributes to a less abrupt cross-linking process, thereby facilitating processing and reducing the occurrence of defects in the composite matrix, the presented results suggest a beneficial practical role for FA as a modifier of PF resin precursors used for composite material matrices. These results suggest that the addition of 30 wt.% FA substantially reduces the viscosity of PF resins, which may facilitate their processing and improve impregnation efficiency without the need for extensive preheating.

## 4. Conclusions

The results provide new insights into the influence of FA on the cross-linking behavior of resol-type PF resins. DRIFTS analysis suggested that the presence of FA influences the distribution of methylene bridge linkages and favors the formation of p-p bridges, with this tendency becoming more pronounced at elevated temperatures. NMR analysis revealed differences in local methylene bridge environments between FA-containing and FA-free systems, supporting the conclusion that FA influences the organization of the cross-linked network. The observed differences in reaction kinetics may also be influenced by the different alkaline catalysts used in the investigated resin systems. STA-FT-IR analysis indicated that FA-containing resins exhibit a more gradual curing process accompanied by a more uniform evolution of volatile products. XPS analysis revealed differences in oxygen-containing functional groups in the cured materials, including a higher contribution of C-O-C and C-OH species in FA-modified resins, consistent with the spectroscopic observations and the proposed differences in curing behavior. Dynamic viscosity measurements demonstrated that the addition of FA substantially reduces the viscosity of PF resins, with viscosities of 144 mPa s and 228 mPa s for FA-containing Resin 2 and Resin 3, respectively, compared with 1051 mPa s and 1104 mPa s for FA-free Resin 1 and Resin 4, respectively. When considered together with our previous findings regarding the extension of gelation time and the reduction of processing defects in PF-based composites, the present results further support the practical potential of FA as a modifier of PF resin systems intended for composite matrix applications. Improved understanding of the role of FA in PF resin curing may support the optimization of resin formulations and processing conditions for advanced composite materials, including carbon-fiber-reinforced polymers and carbon-carbon composites.

## Figures and Tables

**Table 1:** Physicochemical properties of the investigated commercial resins from LERG company, Poland, Pustkow.

Resin Names	Appearance	Non-Volatile Content [wt. %]	Phenol to Formaldehyde Molar Ratio	Free Phenol Content [wt. %]	Free Formaldehyde Content [wt. %]	Catalyst Type	pH at 20 °C
F-110	A clear liquid ranging in color from yellow through cherry-red to dark brown.	>73	1.00:1.29	<15	-	NaOH	7.7–8.5
MC-05	A clear liquid, ranging in color from light brown to dark brown, with no sediment.	74–79	1.00:1.40	<15	<0.1	BaOH_2_	6.7–7.1

**Table 2:** Composition and characteristics of the investigated resin samples.

Sample Name	Type	Resin Content [wt. %]	Furfuryl Alcohol Content [wt. %]	Producer	Product Name
Resin 1	commercial	100	0	LERG	F-110
Resin 2	commercial	70	30	LERG	MC-05
Resin 3	experimental	70	30	LERG	F-110 with FA
Resin 4	experimental	100	0	LERG	MC-05 without FA

**Table 3:** XPS chemical composition of the surface of Resin 1–4.

Element	C				O		Ba	Na
BE [eV]	285.0	286.4	287.9	291.4	532.3	533.3	779.9	1072.4
Groups	C-C C=C(ar.), C-H	C-OH, C-O-C	C=O	π→π* Shake-Up	O=C	O-C, O-H	Ba^2+^	Na^+^
Resin 1	66.9	14.5	0.8	3.0	1.2	13.0	0.0	0.6
Resin 2	60.4	19.1	3.1	2.2	2.3	12.6	0.3	0.0
Resin 3	52.7	20.9	4.9	2.0	4.0	14.7	0.0	0.8
Resin 4	63.5	13.7	3.5	3.2	3.1	12.8	0.2	0.0

## Data Availability

The original contributions presented in this study are included in the article/Supplementary Material. Further inquiries can be directed to the corresponding author.

## Supplementary Materials

The following supporting information can be downloaded at: https://www.mdpi.com/article/10.3390/polym18202513/s1, Figure S1: TGA and DTG curves of (A) Resin 1, (B) Resin 2; Figure S2: (A) DSC and DDSC curves of Resin 1 and (B) DSC, DDSC, and DDDSC curves of Resin 2; Figure S3: DSC, DDSC, and DDDSC curves of Resin 1, maximized to showcase the endothermic peak; Figure S4: (A) DSC and DDSC curves of Resin 2, maximized in the region between 100 and 155 °C, shown in the “horizontal on” mode, (B) DSC, DDSC, and DDDSC curves of Resin 2, maximized in the region between 135 and 190 °C; Figure S5: Gram Schmidt curves of the two resins (red is Resin 1, black is Resin 2); Figure S6: STA-FT-IR 3D plot for Resin 1; Figure S7: STA-FT-IR 3D plot for Resin 2; Figure S8: FT-IR spectrum of products released from Resin 2 at 119 °C, CO_2_, FA (and some water) identified; Figure S9: FT-IR spectrum of products released from Resin 2 at 119 °C, CO_2_, FA, and phenol identified; Figure S10: FT-IR spectrum of products released from Resin 1 at 132 °C, phenol identified; Figure S11: FT-IR spectrum of products released from Resin 1 at 132 °C, furfural and/or some phenol derivative identified; Figure S12: FT-IR spectrum of products released from Resin 2 583 °C water identified; Figure S13: Gas evolution curves for cross-linking process of Resin 1 and Resin 2; Table S1: Summary of the thermal effects observed on the DSC and TGA plots, supplemented with corresponding FTIR information.

## Abbreviations

The following abbreviations are used in this manuscript:

DRIFTS	Diffuse Reflectance Infrared Fourier Transform Spectroscopy
DRS	Diffuse Reflectance Calorimetry
DSC	Differential Scanning Calorimetry
FA	Furfuryl Alcohol
FT-IR	Fourier Transform Infrared Spectroscopy
MS	Mass Spectrometry
MAS-NMR	Magic Angle Spinning—Nuclear Magnetic Resonance
PF	Phenol-Formaldehyde
STA-FT-IR	Simultaneous Thermal Analysis Coupled with Fourier Transform Infrared Spectroscopy
TGA	Thermogravimetric Analysis
XPS	X-ray Photoelectron Spectroscopy
