# Supplementary material for: Influence of Furfuryl Alcohol on the Cross-Linking Process of Selected Industrial Phenol-Formaldehyde Resins
Source: Polymers (Basel). 2026 Oct 14;18(20):2513. doi: 10.3390/polym18202513 (PMC13708871; doi:10.3390/polym18202513)
Supplement: Supplementary file 1 [file polymers-18-02513-s001.zip › polymers-4603587-supplementary.pdf]

Influence of Furfuryl Alcohol on the Cross-Linking Process of Selected Industrial Phenol-Formaldehyde Resins

Maciej Gubernat [1,*], Piotr Jelen [1], Jakub Marchewka [1], Aleksandra Benko [1], Ryszard Wielowski [1], Marcel Zambrzycki [1], Krystian Sokolowski [2] and Aneta Fraczek-Szczypta [1]

[1] Faculty of Materials Science and Ceramics, AGH University of Krakow, Av. Mickiewicza 30, 30-059 Krakow, Poland
[2] Academic Centre for Materials and Nanotechnology, AGH University of Krakow, Av. Mickiewicza 30, 30-059, Krakow, Poland
* Correspondence: maciej.gubernat@agh.edu.pl; Tel.: +48-617-22-39

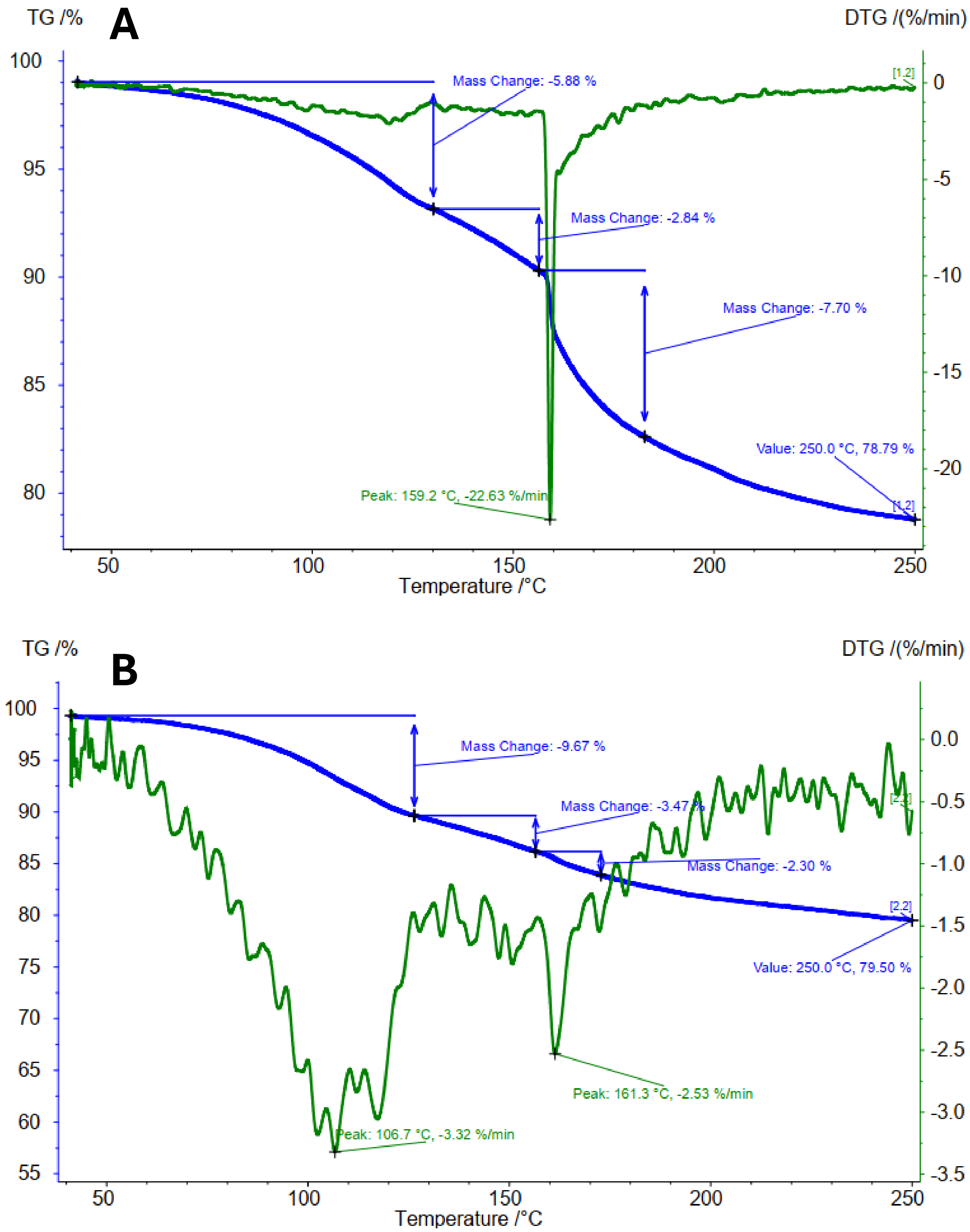

Figure S1 TGA and DTG curves of (A) Resin *1,* (B) Resin 2

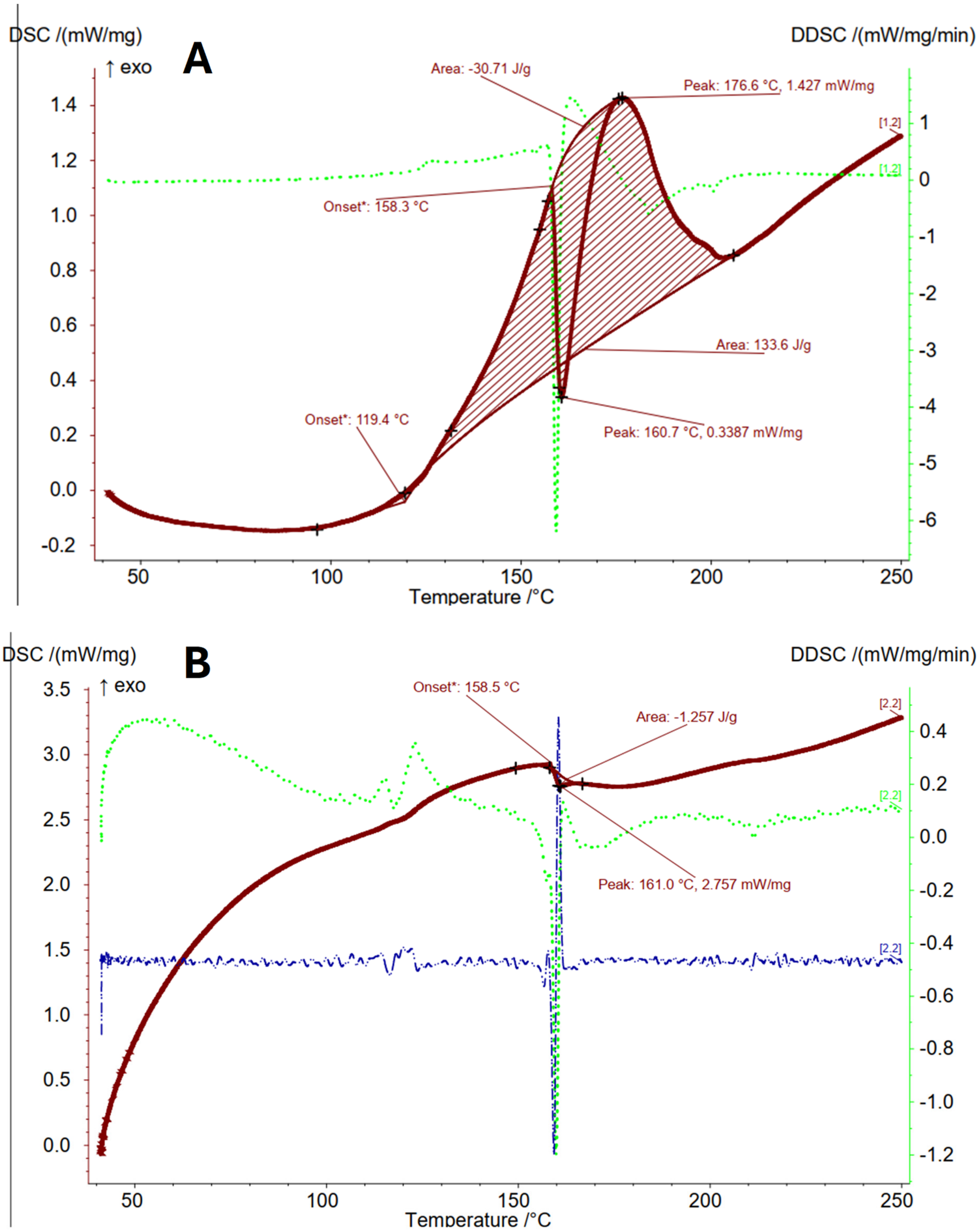

Figure S2 (A) DSC and DDSC curves of Resin 1 and **(B)** DSC, DDSC, and DDDSC curves of Resin 2

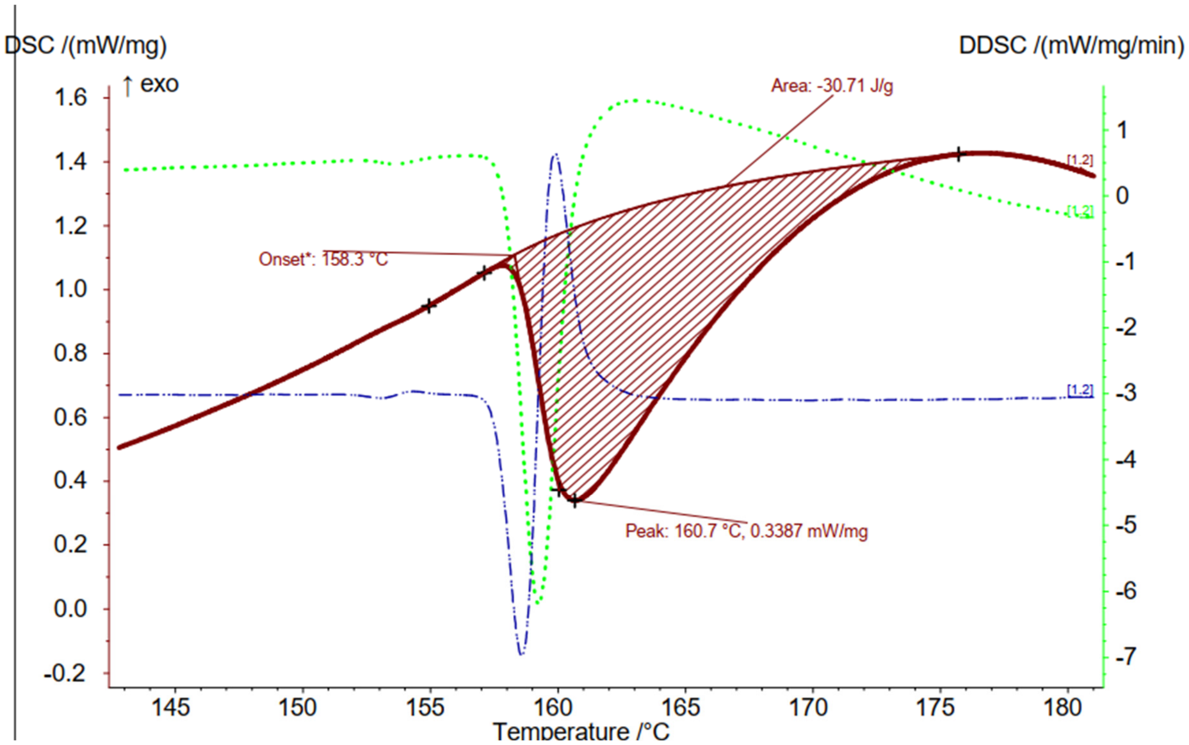

Figure S3 DSC, DDSC, and DDDSC curves of Resin 1, maximized to showcase the endothermic peak

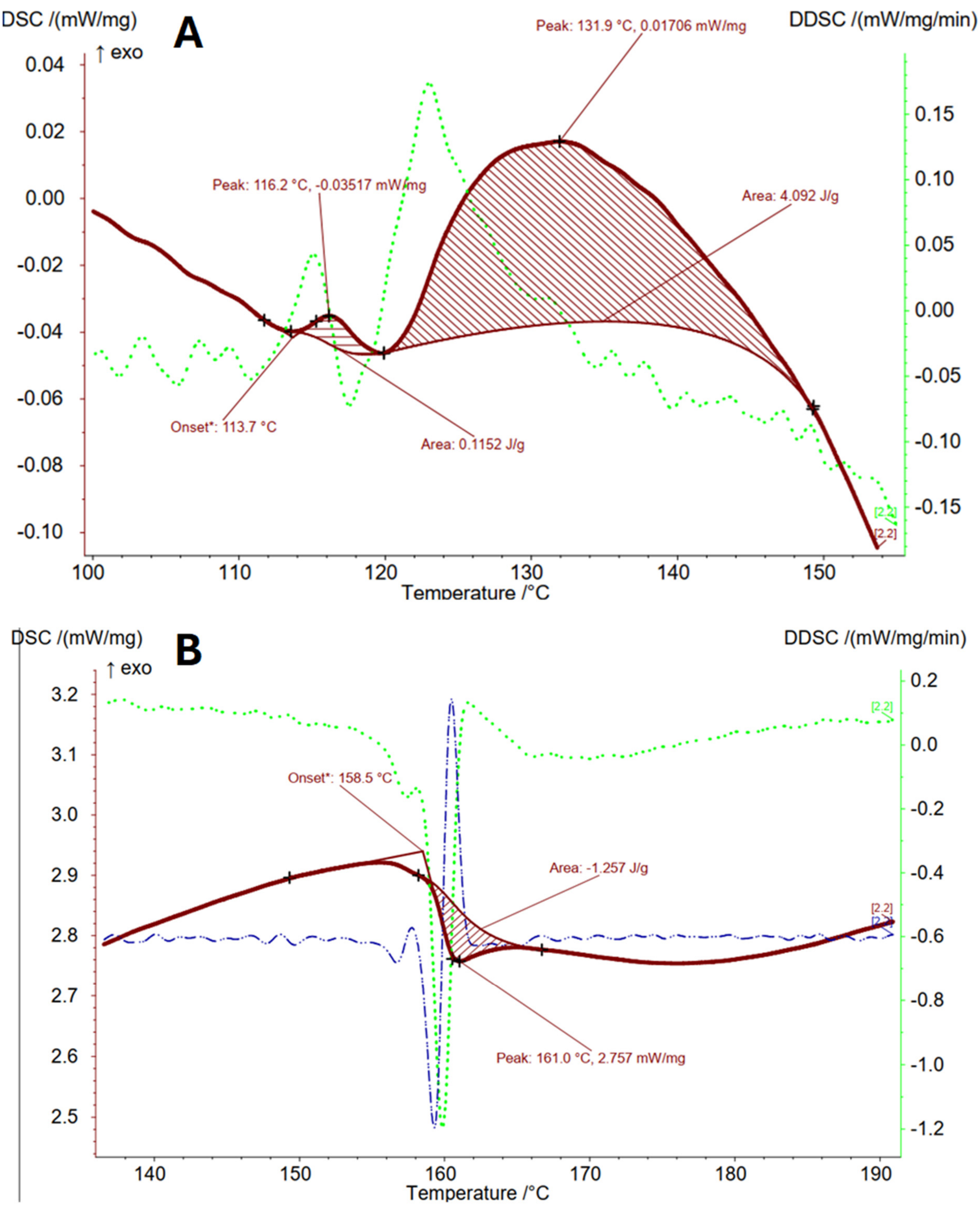

Figure S4 (A) DSC and DDSC curves of Resin 2, maximized in the region between 100 and 155°C, shown in the "horizontal on" mode, **(B)** DSC, DDSC, and DDDSC curves of Resin 2, maximized in the region between 135 and 190 °C

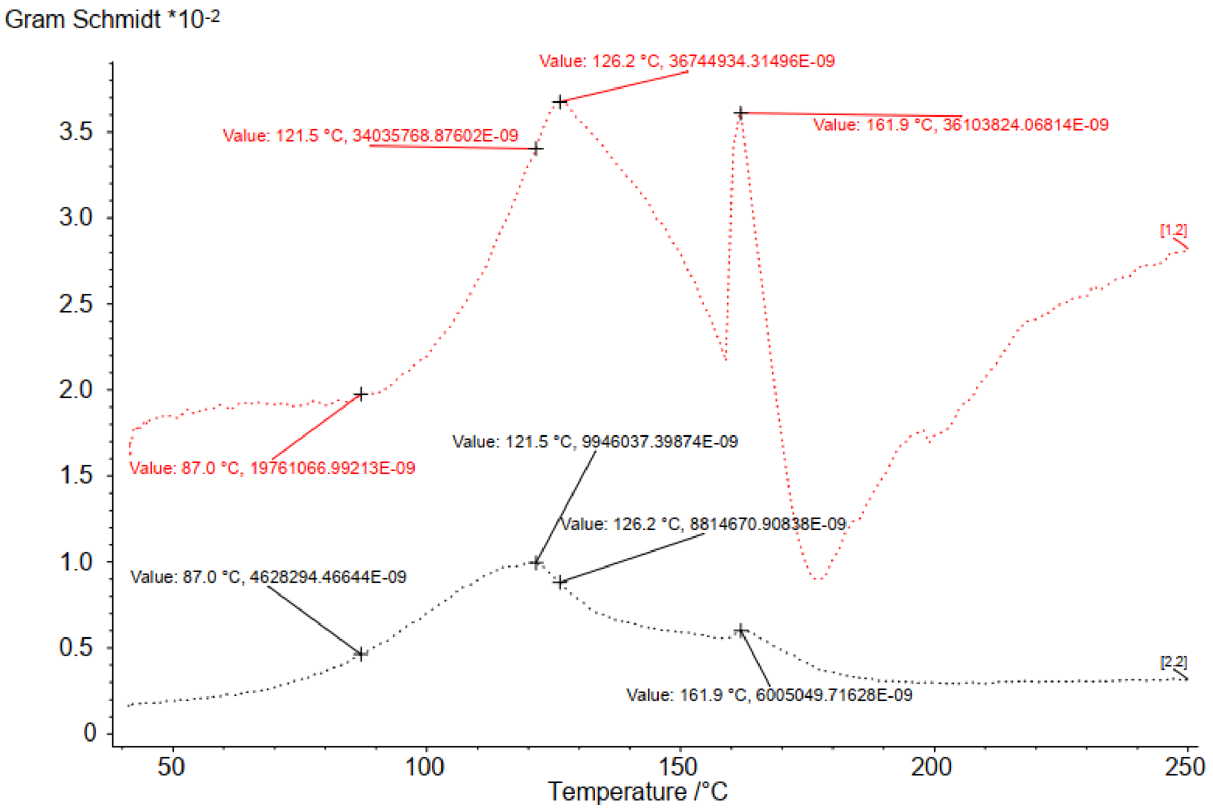

Figure S5 Gram Schmidt curves of the two resins (red is Resin 1, black is Resin 2)

Table S1 Summary of the thermal effects observed on the DSC and TGA plots, supplemented with corresponding FTIR information.

		Resin 1	Resin 2
**Thermal effect 1**	**Temperature range [°C]**	40 - 87	40 - 70
	**Energy change (positive is exo)**	0	1.7 **[mW/mg]**
	**Mass loss**	- 3 %	- 2 %
	**Main volatile products**	CO, methane	CO_2, FA
**Thermal effect 2**	**Temperature range [°C]**	87 - 119	70 - 114
	**Energy change**	0	0.7 **[mW/mg]**
	**Mass loss**	- 2%	- 7%
	**Main volatile products**	methane, water	CO_2, FA, phenol, methane
**Thermal effect 3 – exothermal peak**	**Temperature range [°C]**	119 - 206	114 – 149
	**Energy change**	113.6 J/g	4.2 J/g
	**Mass loss**	- 14%	- 5%
	**Main volatile products**	CO, phenol, methane, water	CO_2, FA, phenol, methane
	**Suggested reaction**	One-step curing	Two-step curing (initial condensation of methylol groups, followed by conversion into methylene bridges.)
**Thermal effect 4 – endotherm al peak**	**Temperature range [°C]**	158 - 177 (superimposed on effect no. 3)	158 – 182 (separate from effect no. 3)
	**Energy change**	-30.7 J/g	- 1.3 J/g
	**Mass loss**	- 8%	- 3%
	**Main volatile products**	CO, phenol, methane	CO_2, water
	**Suggested reaction**	Evaporation of by-products	Evaporation of by-products
**Thermal effect 5**	**Temperature range [°C]**	206 - 250	182 - 250
	**Energy change**	0.5 **[mW/mg]**	0.6 **[mW/mg]**
	**Mass loss**	- 2%	- 3%
	**Main volatile products**	CO, phenol,	CO_2, water

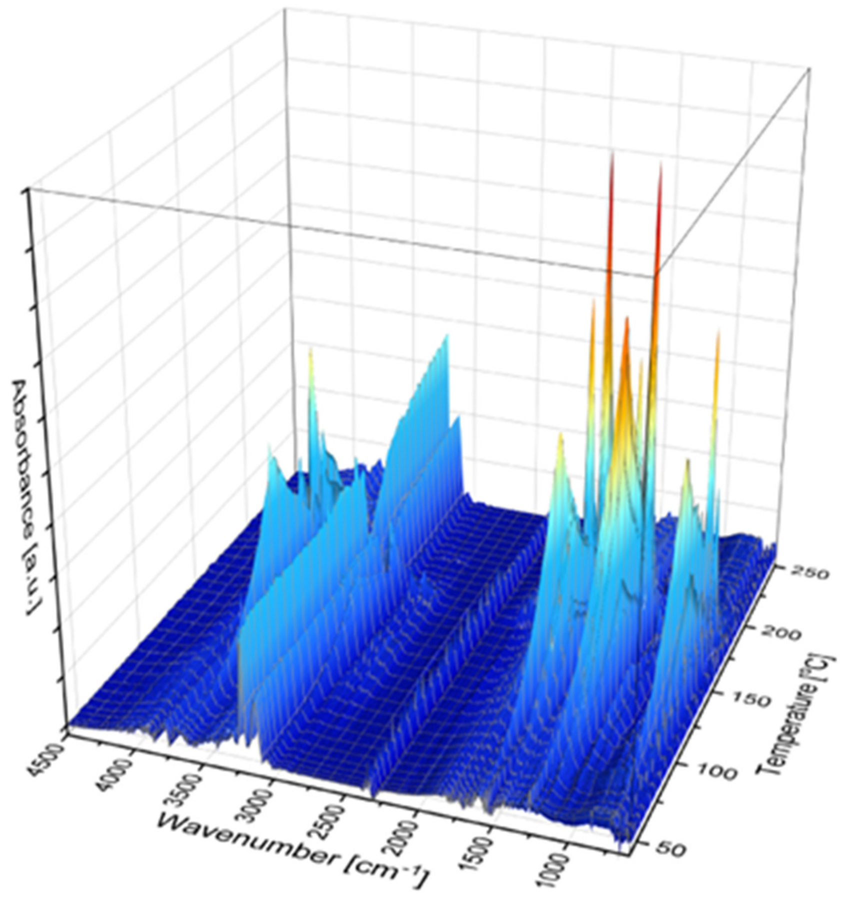

Figure S6 STA-FT-IR 3D plot for Resin 1

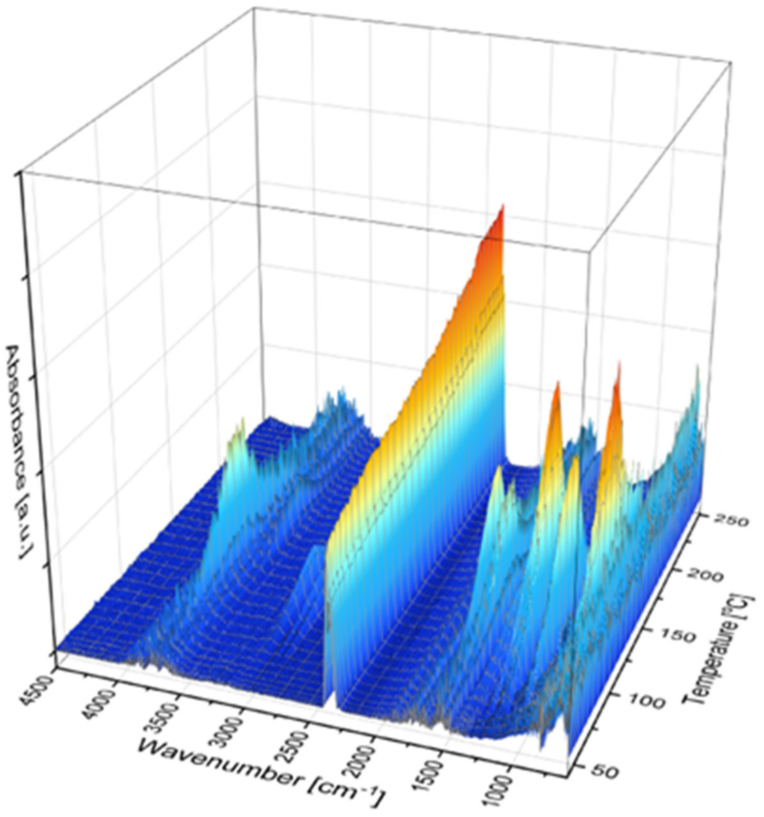

Figure S7 STA-FT-IR 3D plot for Resin 2

OPUS/SEARCH 10/10/2023

C:\OPUS_7.2.139.1294\Data\TG_FTIR\2022_03_14\select\2023_09_06_LERG M110powtorzenie_1000_azot.0_AB_162_119.863998_Temperature.0 M 4.7 | NETZSCH STA 449F3 | C:\NETZSC

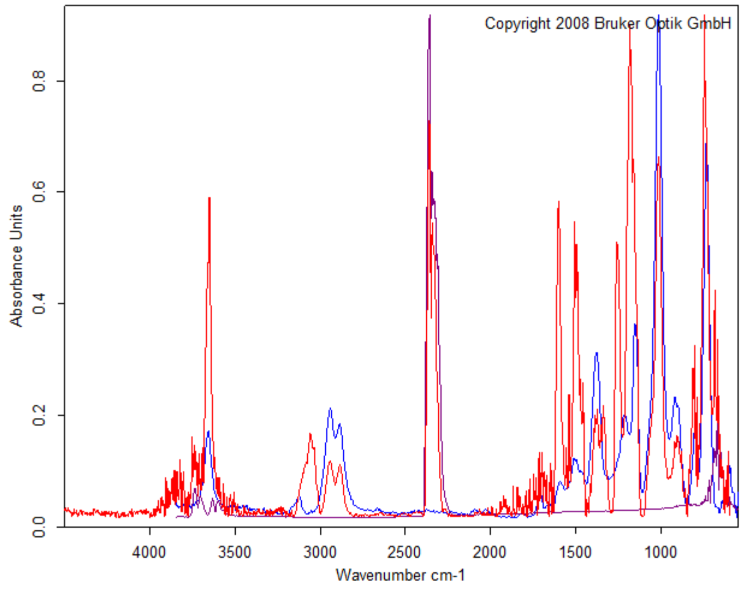

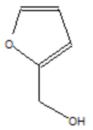

Compound information

Compound Name	Furfurylalcohol
Molecular Formula	C5H6O2
Molecular Weight	98.1
CAS Registry Number	98-00-0
Manufacturer	NIST Mass Spectrometry Data Center

Carbon dioxide

Furfurylalcohol

Hit No.	Hit Quality	Compound Name	Entry No.	Lib. Index	Molecular formula	Molecular weight	CAS number
1	462	Benzotriazole	3016	2	C6H5N3	119.13	95-14-7
2	452	Carbon dioxide	3233	2	C1O2	44.01	124-38-9
3	446	9-Hydroxyfluorene	2205	2	C13H10O1	182.22	1689-64-1
4	430	.alpha.-Chloroacetophenone	34	2	C8H7Cl1O1	154.6	532-27-4
5	417	Furfurylalcohol	3915	2	C5H6O2	98.1	98-00-0

Figure S8 FT-IR spectrum of products released from Resin 2 at 119 °C, CO_2, FA (and some water) identified

OPUS/SEARCH **10/10/2023**

C:\OPUS_7.2.139.1294\Data\TG_FTIR\2022_03_14\select\2023_09_06_LERG M110powtorzenie_1000_azot.0_AB_162_119.863998_Temperature.0 M 4.7 | NETZSCH STA 449F3 | C:\NETZSC

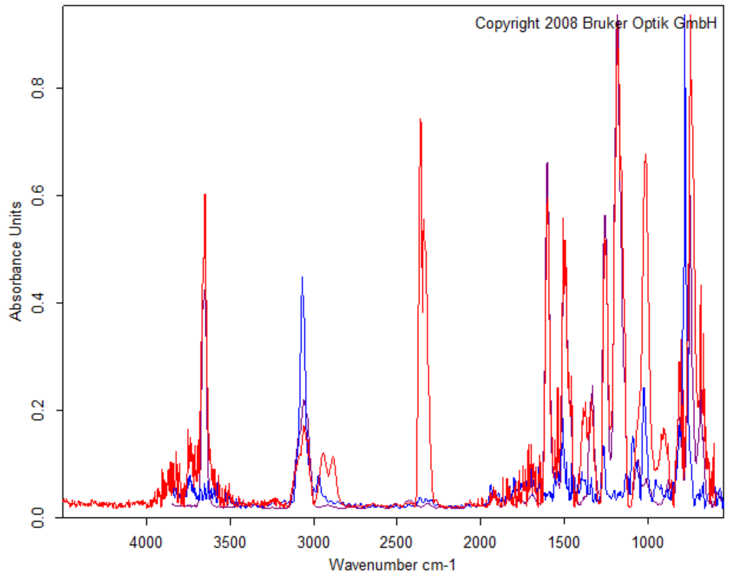

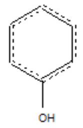

Compound information	
Compound Name	Phenol
Molecular Formula	C6H6O1
Molecular Weight	94.11
CAS Registry Number	108-95-2
Melting Point	39
Boiling Point	181
Manufacturer	Sadtler Research Labs Under US-EPA Contrac

1-Naphthalenesulfonic acid

Phenol

Hit No.	Hit Quality	Compound Name	Entry No.	Lib. Index	Molecular formula	Molecular weight	CAS number
1	365	1-Naphthalenesulfonic acid	573	2	C10H8O3S1	208.24	85-47-2
2	309	Phenol	4692	2	C6H6O1	94.11	108-95-2
3	265	Benzenamine, 4-chloro-2-nitro-N-phenyl-	2491	2	C12H9Cl1N2O2	248.67	16611-15-7
4	258	Phenol, 4,4'-(1-methylethylidene)bis-	4668	2	C15H16O2	228.29	80-05-7
5	255	Carbon dioxide	3233	2	C1O2	44.01	124-38-9

Figure S9 FT-IR spectrum of products released from Resin 2 at 119 °C, CO_2, FA, and phenol identified

OPUS/SEARCH **10/10/2023**

C:\OPUS_7.2.139.1294\Data\TG_FTIR\2022_03_14\select\2023_09_08_LERG F_1000powtorzenie_azot.0_AB_170_132.662003_Temperature.0 f 13.8 | NETZSCH STA 449F3 | C:\NETZSCH\F

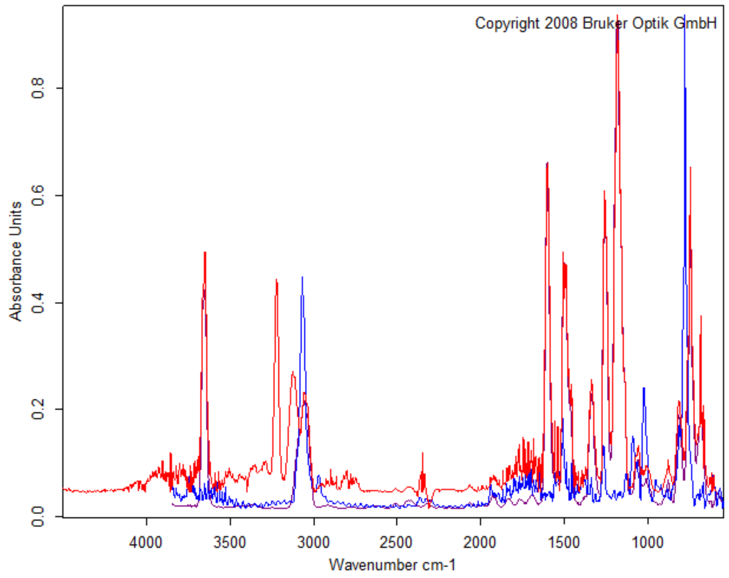

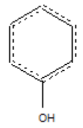

Compound information	
Compound Name	Phenol
Molecular Formula	C6H6O1
Molecular Weight	94.11
CAS Registry Number	108-95-2
Melting Point	39
Boiling Point	181
Manufacturer	Sadtler Research Labs Under US-EPA Contrac

1-Naphthalenesulfonic acid

Phenol

Hit No.	Hit Quality	Compound Name	Entry No.	Lib. Index	Molecular formula	Molecular weight	CAS number
1	205	1-Naphthalenesulfonic acid	573	2	C10H8O3S1	208.24	85-47-2
2	193	Phenol	4692	2	C6H6O1	94.11	108-95-2
3	154	Phenol, 4,4'-(1-methylethylidene)bis-	4668	2	C15H16O2	228.29	80-05-7

Figure S10 FT-IR spectrum of products released from Resin 1 at 132 °C, phenol identified

OPUS/SEARCH 1/31/2024

C:\OPUS_7.2.139.1294\Data\TG_FTIR\2022_03_14\select\f110\2023_09_08_LERG F_1000powtorzenie_azot.0_AB_170_132.662003_Temperature.0 f 13.8 | NETZSCH STA 449F3 | C:\NETZS

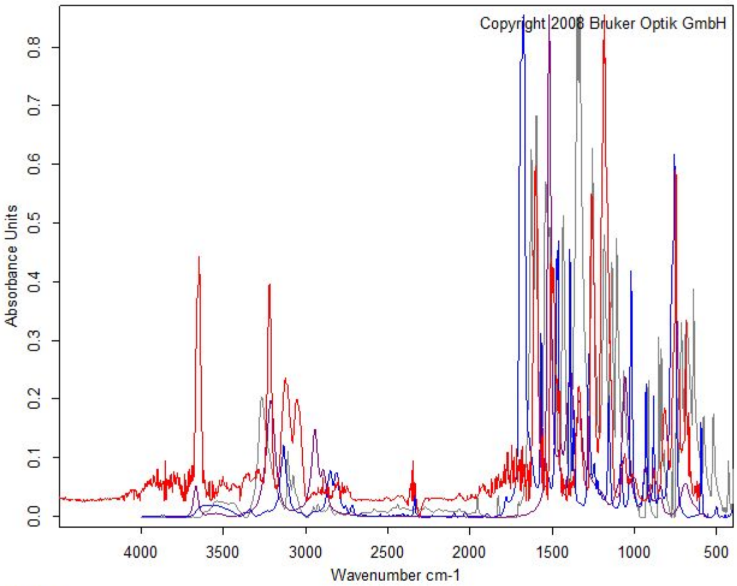

Compound information
Compound Name 2,4-DINITROPHENOL
Molecular Formula C6H4N2O5
Molecular Weight 184.1
CAS Registry Number 51-28-5
Melting Point 113
Sample Preparation KBR 0.00075 G/CM^2

FURFURAL

N,N'-Bis(2-hydroxyethyl)dithioxamide

2,4-DINITROPHENOL

Hit No.	Hit Quality	Compound Name	Entry No.	Lib. Index	Molecular formula	Molecular weight	CAS number
1	502	PROPYLAMINE	19	4			
2	494	2-Amino-2-methyl-1-propanol	222	3	C4H11N1O1	89.14	124-68-5
3	404	1,7-DIAMINOHEPTANE	186	1	C7H18N2	130.23	646-19-5
4	385	(n-Butylamino)acetonitrile	1	2	C6H12N2	112.18	3010-04-6
5	381	Picolinamide, N-(1,1,3,3-tetramethylbutyl)-5-ethylthio-	4737	2	C16H26N2S1	278.46	70289-18-8

Figure S11 FT-IR spectrum of products released from Resin 1 at 132 ^{o}C, furfural and/or some phenol derivative identified

OPUS/SEARCH **10/10/2023**

C:\OPUS_7.2.139.1294\Data\TG_FTIR\2022_03_14\select\2023_09_06_LERG M110powtorzenie_1000_azot.0_AB_542_582.718018_Temperature.0 M 4.7 | NETZSCH STA 449F3 | C:\NETZSC

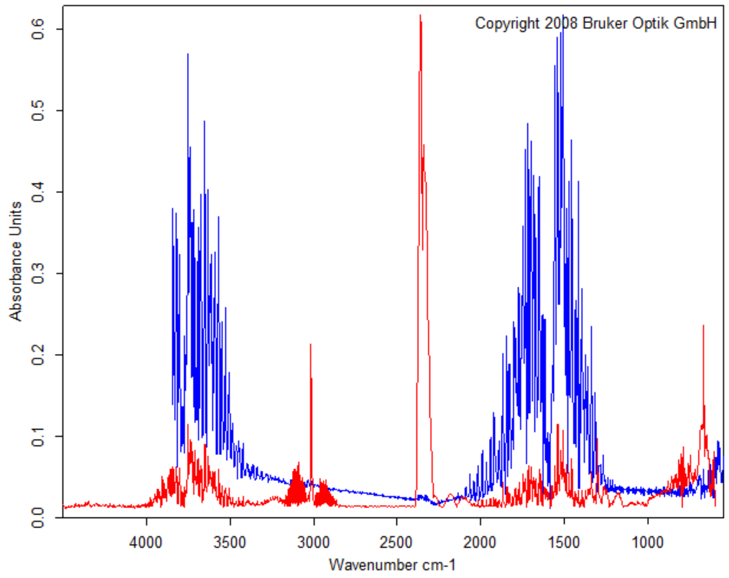

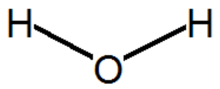

Compound information

Compound Name	Water
Molecular Formula	H2O1
Molecular Weight	18.02
CAS Registry Number	7732-18-5
Manufacturer	Sadtler Research Labs Under US-EPA Contrac

Water

Hit No.	Hit Quality	Compound Name	Entry No.	Lib. Index	Molecular formula	Molecular weight	CAS number
1	643	Water	5229	2	H2O1	18.02	7732-18-5
2	357	Deuterium oxide	3534	2	D2O1		7789-20-0
3	234	Methane, bromo-	4154	2	C1H3Br1	94.94	74-83-9
4	209	Codeine	67	3	C18H21N1O3*H2O1	317.39	
5	207	Benzoic acid, 3,5-dinitro-, isobutyl ester	2950	2	C11H12N2O6	268.23	10478-01-0

Figure S12 FT-IR spectrum of products released from Resin 2 583 °C water identified

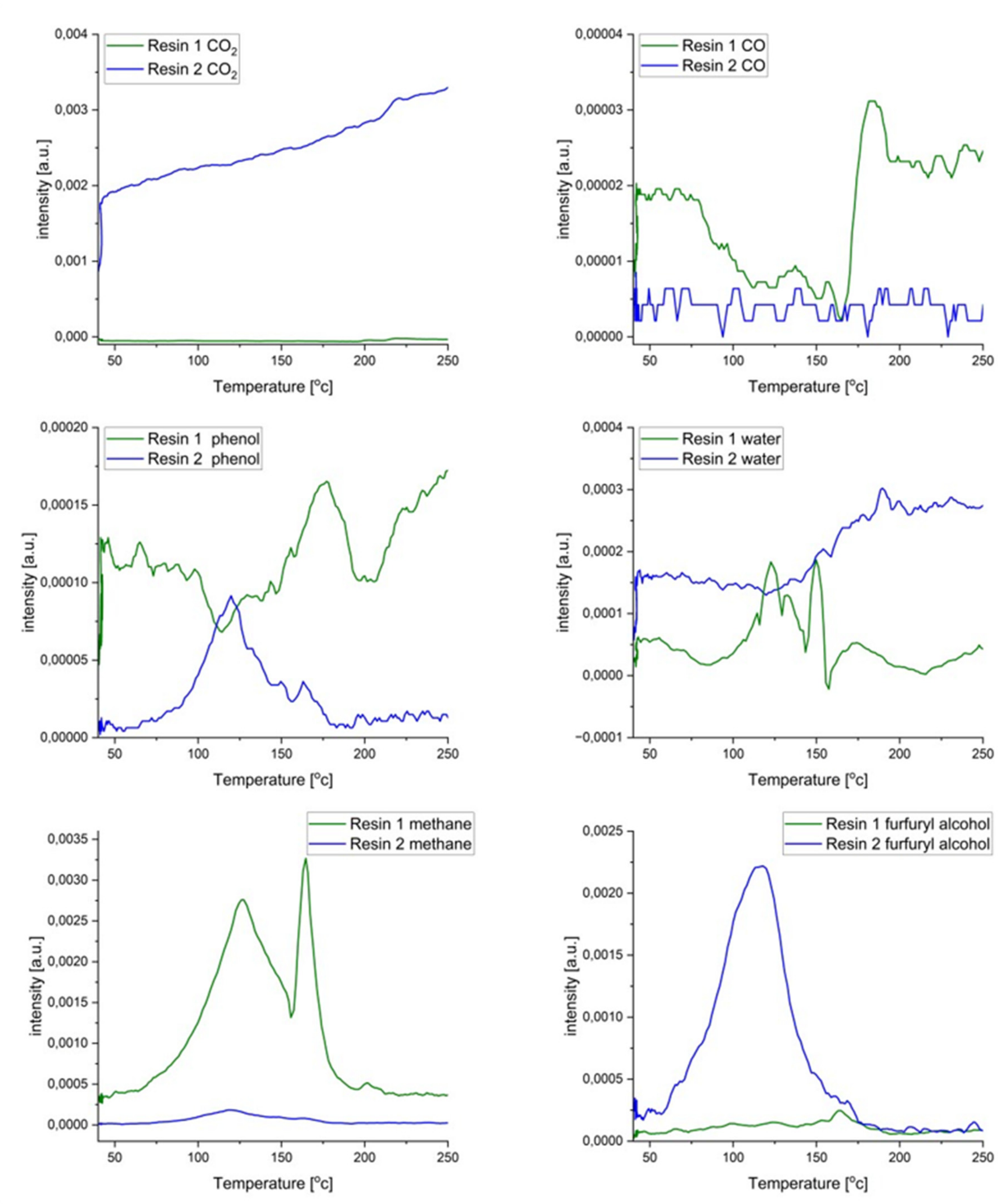

Figure S13 Gas evolution curves for cross-linking process of Resin 1 and Resin 2
